# From Sequence to Response: AI‐Guided Prediction of Nucleic Acid Nanoparticles Immune Recognitions

**DOI:** 10.1002/smll.202509459

**Published:** 2025-10-28

**Authors:** M. Brittany Johnson, Sankalp Jain, Jessica McMillan Shea, Quinton Krueger, Erwin Doe, Daniel Miller, Katelynn Pranger, Hannah Hayth, Sable Thornburgh, Justin Halman, Emil F. Khisamutdinov, Alexey V. Zakharov, Kirill A. Afonin

**Affiliations:** ^1^ Department of Biological Sciences University of North Carolina at Charlotte 9201 University City Boulevard Charlotte NC 28223 USA; ^2^ National Center for Advancing Translational Sciences National Institutes of Health Rockville MD 20850 USA; ^3^ Nanoscale Science Program Department of Chemistry University of North Carolina at Charlotte Charlotte NC 28223 USA; ^4^ Computational Intelligence for Predicting Health and Environmental Risks (CIPHER) University of North Carolina at Charlotte Charlotte NC 28223 USA; ^5^ Department of Chemistry Ball State University Muncie IN 47306 USA

**Keywords:** artificial intelligence, immune stimulation, new approach methodologies (NAMs), nucleic acid nanoparticles (NANPs), quantitative structure‐activity relationship (QSAR), microglia

## Abstract

Nucleic acid nanoparticles (NANPs) represent a versatile platform for drug delivery and modulation of therapeutic responses. To expedite NANPs’ translation from bench to bedside, rapid coordination of their design principles with immunostimulatory assessment is essential. Here, a deep learning framework is presented to predict cytokine responses, specifically interferon‐beta (IFN‐β) and interleukin‐6 (IL‐6), induced by NANPs in human microglial cells based solely on their sequences. Using a transformer‐based architecture augmented through systematic strand permutation trained on 176 structurally diverse, individually assembled, and experimentally characterized NANPs, the model achieved high predictive performance in cross‐validation (R^2^ = 0.96–0.97, RMSE ≤ 0.01) and demonstrated strong generalizability on an external test set (R^2^ = 0.91 for IFN‐β; 0.85 for IL‐6). This work advances sequence‐based quantitative structure‐activity relationship (QSAR) modeling by leveraging attention‐based neural networks to eliminate the need for manual feature engineering while maintaining biological interpretability. To facilitate community access, the updated artificial immune cell (AI‐cell) web‐based platform is introduced, which supports rapid immune profiling of NANPs *in silico*. This new approach methodology provides a scalable framework to guide the rational design and optimization of NANPs through rapid prediction of their immune responses.

## Graphical abstract

We introduce a transformer‐based deep learning framework that predicts cytokine responses triggered by NANPs, enabling their rapid and scalable immune profiling through the A‐Icell web platform for therapeutic design and optimization.

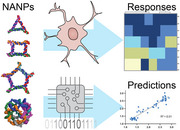

## 1 Introduction

Therapeutic nucleic acids (TNAs) have gained attention for their potential in targeted drug delivery and immunotherapy.^[^ ^1^ ^]^ Despite recent advances, TNAs still face challenges in achieving personalized formulations involving multiple therapeutic agents with precisely regulated immunorecognition and toxicity. Nucleic acid nanoparticles (NANPs) offer enhanced control over therapeutic composition and architectural parameters of TNAs while allowing customization to match the specific immunological response profile of a patient.^[^ ^2^, ^3^, ^4^, ^5^ ^]^ NANPs are composed of nucleic acid strands that are designed to self‐assemble into well‐defined nanoscale structures. As such, NANPs can be engineered for high chemical and thermodynamic stability, multifunctionality, and tunable immunological properties, ranging from immunoquiescent to immunostimulatory, depending on the intended biomedical application. For example, previously reported planar polygonal^[^ ^6^, ^7^ ^]^ and globular^[^ ^5^, ^6^, ^7^, ^8^, ^9^, ^10^, ^11^, ^12^, ^13^, ^14^ ^]^ NANPs offer an ideal model system for probing structure‐function relationships due to their symmetry, tunable valency, and well‐defined inter‐strand interactions. The tunable architectural characteristics make these representative NANPs suitable for exploring the influence of dimensionality, strand composition, and structural complexity on immune recognition.

Assessing the immunological properties of all newly engineered NANPs is a time‐consuming process that requires specialized training and equipment. Therefore, the development of new approach methodologies (NAMs), such as computational models capable of rapidly predicting the immunorecognition of NANPs, would greatly accelerate progress in the field and deepen our mechanistic understanding of NANP‐mediated immune responses. The implementation of these NAMs would also support the establishment of standards to assist the global nucleic acid nanotechnology community in translating their technologies to the clinical stage.

We developed the first computational approach that predicts the immunological properties of NANPs delivered to freshly isolated human peripheral blood mononuclear cells (PBMCs).^[^ ^13^ ^]^ The PBMC model was chosen for its greater accuracy in predicting cytokine storm toxicity compared to conventional preclinical animal models such as rodents and primates.^[^ ^15^, ^16^, ^17^ ^]^ Through continuous feedback between physicochemical characterization of ≈60 representative NANPs, their detailed immunological profiling, and machine learning, we introduced a new web‐based tool, called “Artificial Immune Cell” or “AI‐cell”. AI‐cell predicts immune responses for input NANP sequences in just 2–4 s, compared to at least 48 h required for experimental assays.^[^ ^18^ ^]^

**Figure 1:**
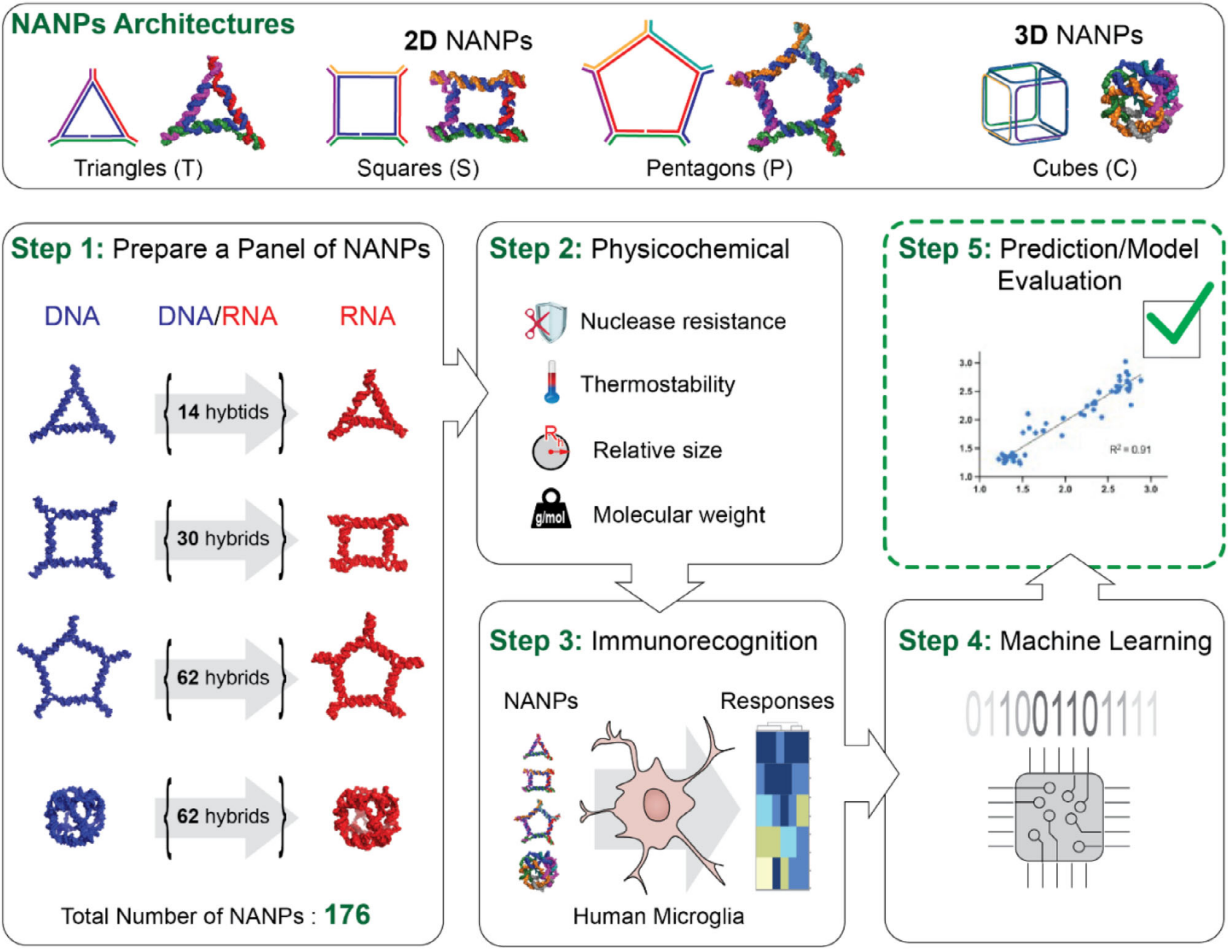
Schematic of the experimental flow. The upper panel illustrates the connectivity principles of selected NANP architectures. The lower panels depict the experimental workflow, including the production of a diverse NANP library, and side‐by‐side physicochemical characterization of all NANPs with further assessment of their immunorecognition using in vitro biological assays, application of machine learning to model immune responses based on structural and compositional features, and validation of the developed model.

To expand the utility of AI‐cell and enable rapid, accurate prediction of NANP immunorecognition across diverse therapeutic contexts, including interactions with various immune cell subpopulations, we now develop predictive models for NANP‐induced immune responses in human microglial cells, a clinically relevant innate immune cell type implicated in neuroinflammation and CNS‐specific cytokine release. Microglia, as the primary immune sentinels of the central nervous system, play a pivotal role in neuroinflammatory processes implicated in disorders ranging from neurodegenerative diseases to glioblastoma. Understanding how NANPs interact with microglia is therefore critical for the safe and effective application of nucleic acid therapeutics in CNS‐targeted strategies. To evaluate the influence of various architectural parameters and chemical compositions of NANPs, we conducted systematic studies using a comprehensive panel of 112 planar and 64 globular NANPs composed of short RNA and DNA strands (**Figure** **1**). These NANPs were designed to self‐assemble into well‐defined architectures, including 2D four‐stranded triangles, five‐stranded squares, six‐stranded pentagons, and 3D six‐stranded cubes. For each NANP, we characterized physicochemical properties such as size, melting temperature, and chemical stability in serum, and assessed their immunostimulatory profiles in human microglia. A transformer‐based model was trained and validated to reliably predict immune responses across this diverse set of NANP sequences.

Our current study represents the most comprehensive evaluations to date of NANP‐induced immune responses in human microglia, combining structural diversity, high‐throughput biological assessment, and state‐of‐the‐art machine learning in a unified workflow. This work builds on recent advances in artificial intelligence (AI) and machine learning (ML) that are increasingly transforming the design and functional prediction of complex biomaterials, including NANPs.^[^ ^6^, ^13^, ^19^, ^20^, ^21^ ^]^ Transformer‐based models have shown notable advantages over traditional ML approaches, especially for capturing sequence‐level patterns that underlie biological function.^[^ ^22^, ^23^ ^]^ By applying systematic data augmentation and rigorous model validation, including external test set evaluation, we demonstrate the robustness and generalizability of the current models across diverse NANP architectures. This framework enables data‐driven design of next‐generation nanotherapeutics and reduces the reliance on time‐ and resource‐intensive experimental screening.

To further support broad adoption and community engagement, the top‐performing models from the current study are now publicly available at https://aicell.ncats.io. The enhanced interactive interface allows users to predict immune responses of novel NANPs and TNAs directly from sequence input, enabling data‐driven design of next‐generation TNAs. We also included representative control NANPs that have the lowest and the highest immune recognition.

Together, these advancements mark an important step toward predictive, sequence‐driven immunological profiling of NANPs for therapeutic applications. Our new approach methodology aligns with the FDA Modernization Act 2.0, a bipartisan initiative to reduce reliance on animal testing and promote innovative science. Broad implementation of this NAM could streamline NANP‐based drug development, making it more ethical, efficient, and reliable. Moreover, the developed modeling framework can be extended to other therapeutic modalities, including biologics and small molecules.

## 2 Results

### 2.1 NANPs have Predictable and Tunable Physicochemical Properties

In nucleic acid nanotechnology, physicochemical characterization is essential for understanding and optimizing the structural integrity, stability, and functional properties of NANPs.^[^ ^5^, ^6^, ^7^, ^8^, ^10^, ^24^ ^]^ Due to their complex and programmable architecture, NANPs pose unique challenges for characterization, as factors such as chemical stability, size, shape, and thermal behavior directly impact their biological activity, biocompatibility, and delivery efficiency. Comprehensive characterization is therefore critical for tuning NANPs for specific applications in therapeutics, diagnostics, and biosensing and to inform the rational design of NANPs for reliable performance in complex biological systems.

**Figure 2:**
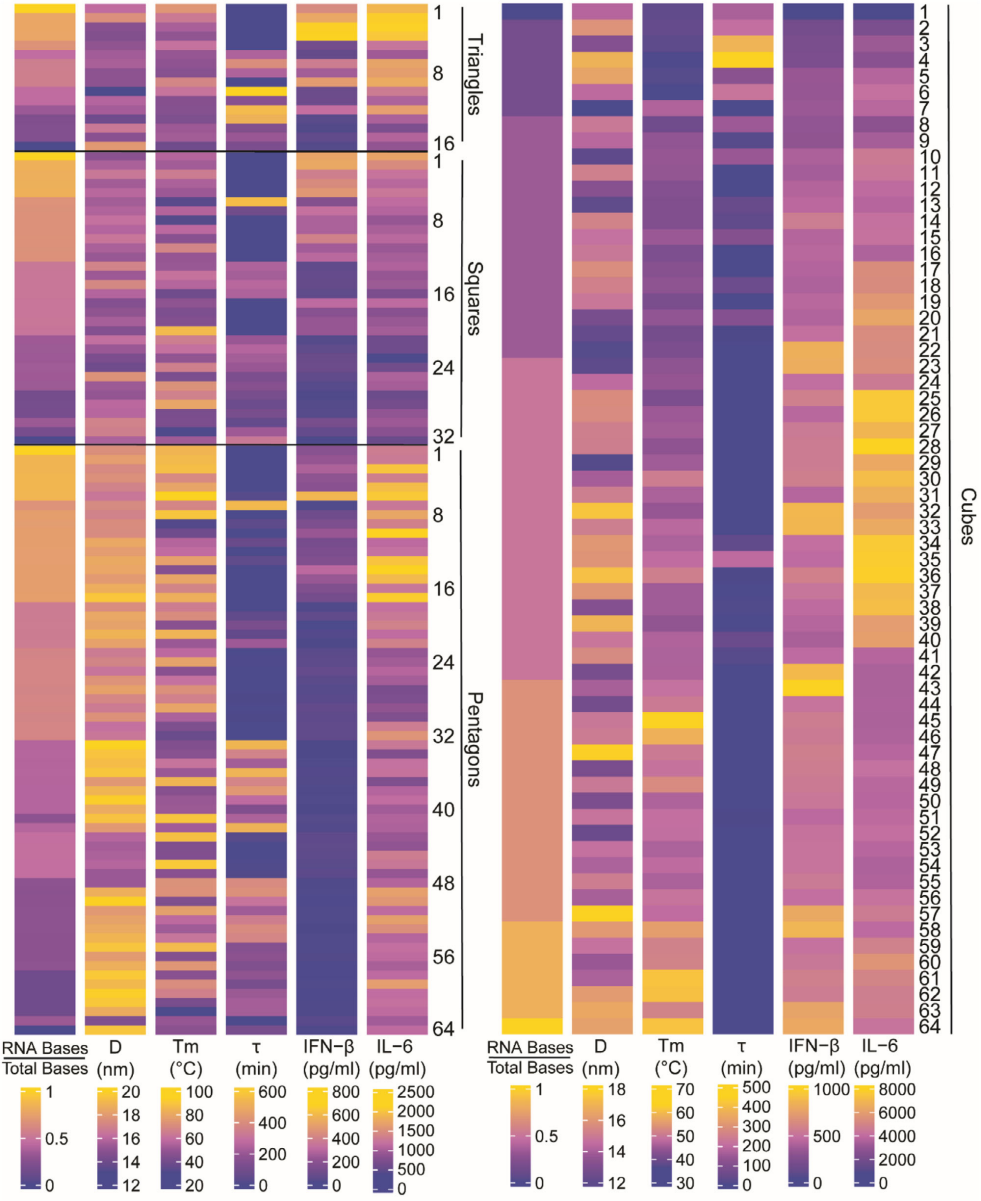
Heatmaps of 2D (left) and 3D (right) NANPs. The raw values of the ratio of RNA bases to total bases, diameter (D), melting temperature (T_m_), serum stability (τ), IFN‐β production, and IL‐6 production for all NANPs were drawn on independent scales across each variable. NANP triangles, squares, and pentagons are abbreviated Triangles 1–16, Squares 1–32, and Pentagons 1–64, respectively. NANP cubes are abbreviated as Cubes 1–64. In this NANP series, Triangle 1, Square 1, Pentagon 1, and Cube 64 are composed entirely of RNA strands, while Triangle 16, Square 32, Pentagon 64, and Cube 1 are composed entirely of DNA strands. Data are presented as the mean of at least three independent replicates.

To characterize the physicochemical properties of our panel of 176 representative NANPs, we employed a suite of analytical techniques. Electrophoretic mobility shift assays (EMSA) were employed to confirm the correct formation of NANPs. Dynamic light scattering (DLS) was used to determine NANPs size distributions and assess the polydispersity index (PDI), with results indicating moderate monodispersity for triangular, square, and pentagonal NANPs (PDI = 0.3–0.6) and lower PDI for cubic NANPs (PDI 0.1‐0.3). All NANPs showed an average diameter of ≈15 nm, with a general trend of increasing diameter as the number of DNA strands in the construct increased (**Figures** **2** and S2–S4, Supporting Information). UV‐melting curves provided insights into the thermal stability of each structure, revealing characteristic melting temperatures (T_m_) for triangular, square, pentagonal, and cubic forms. This thermal stability analysis was conducted across a series of NANPs (16 triangular, 32 square, 64 pentagonal, and 64 cuboidal) as demonstrated in Figures 2 and S5–S7 (Supporting Information). For triangular NANPs, two distinct thermal transitions were observed, with the first occurring ≈50 °C and the second ≈75 °C. Notably, a general trend was observed in all NANPs such that the T_m_ values decreased as the number of DNA strands increased. The T_m_ was highest for particles composed entirely of RNA (T1, S1, P1, C64), while complexes constructed solely from DNA (T16, S32, P64, and C1) were the least stable. This decreasing T_m_ trend correlates with structural composition, which also reflects the stability hierarchy among the nucleic acid components.^[^ ^5^, ^6^, ^7^, ^8^, ^25^, ^26^ ^]^

In the biological stability assay, differential resistance to nuclease degradation was observed among the various NANP structures Figures 2 and S8 and S9 (Supporting Information). Hybrid complexes, composed of both RNA and DNA, demonstrated the highest resistance to nuclease activity, maintaining structural integrity over extended incubation times. Specifically, NANP structures T10, T12, T13, S6, P7, P33, P36, P42, C3, and C4 displayed the highest stability. This suggests that the hybrid composition may confer added stability by balancing the biochemical properties of both nucleic acids. DNA‐only NANPs showed moderate stability, displaying increased degradation compared to hybrid structures but greater resistance than RNA‐only analogs. RNA‐only NANPs were the least stable (degraded within 1 min), showing significant degradation under the same conditions, which is consistent with the generally higher susceptibility of RNA to nucleases in serum.^[^ ^27^, ^28^, ^29^, ^30^, ^31^ ^]^ This stability hierarchy highlights the advantage of hybrid NANPs in applications requiring enhanced nuclease resistance, such as in vivo delivery or prolonged circulation in biological environments.

### 2.2 NANPs have Predictable and Programmable Immunostimulatory and Immunoquiescent Properties

Due to the severity of central nervous system diseases and the difficulty in delivering therapeutics across the blood‐brain barrier, there is significant interest in the exploration of nanoparticles as therapeutics for the treatment of CNS diseases.^[^ ^32^, ^33^, ^34^, ^35^, ^36^, ^37^ ^]^ Nanoparticles have the significant advantage of being able to readily cross the blood‐brain barrier. However, one of the main challenges to employing TNAs within the CNS is the rational design of nanoparticles with defined and desired immunostimulatory or immunoquiescent properties. It is now appreciated that tissue resident cells of the brain parenchyma, such as microglia, contribute to the regulation of brain development and tissue homeostasis. These macrophage‐like cells provide important immune surveillance to the brain, which is often considered immune‐privileged due to the blood‐brain barrier.^[^ ^38^, ^39^, ^40^, ^41^, ^42^, ^43^ ^]^ Notably, it is now recognized that microglia express pattern recognition receptors (PRRs) that can recognize nucleic acid ligands and initiate innate immune responses.^[^ ^41^, ^42^, ^43^, ^44^ ^]^ As such, microglia are a relevant model for assessing the immunological profiles of novel formulations that may be employed in the treatment of neurological diseases.

**Figure 3:**
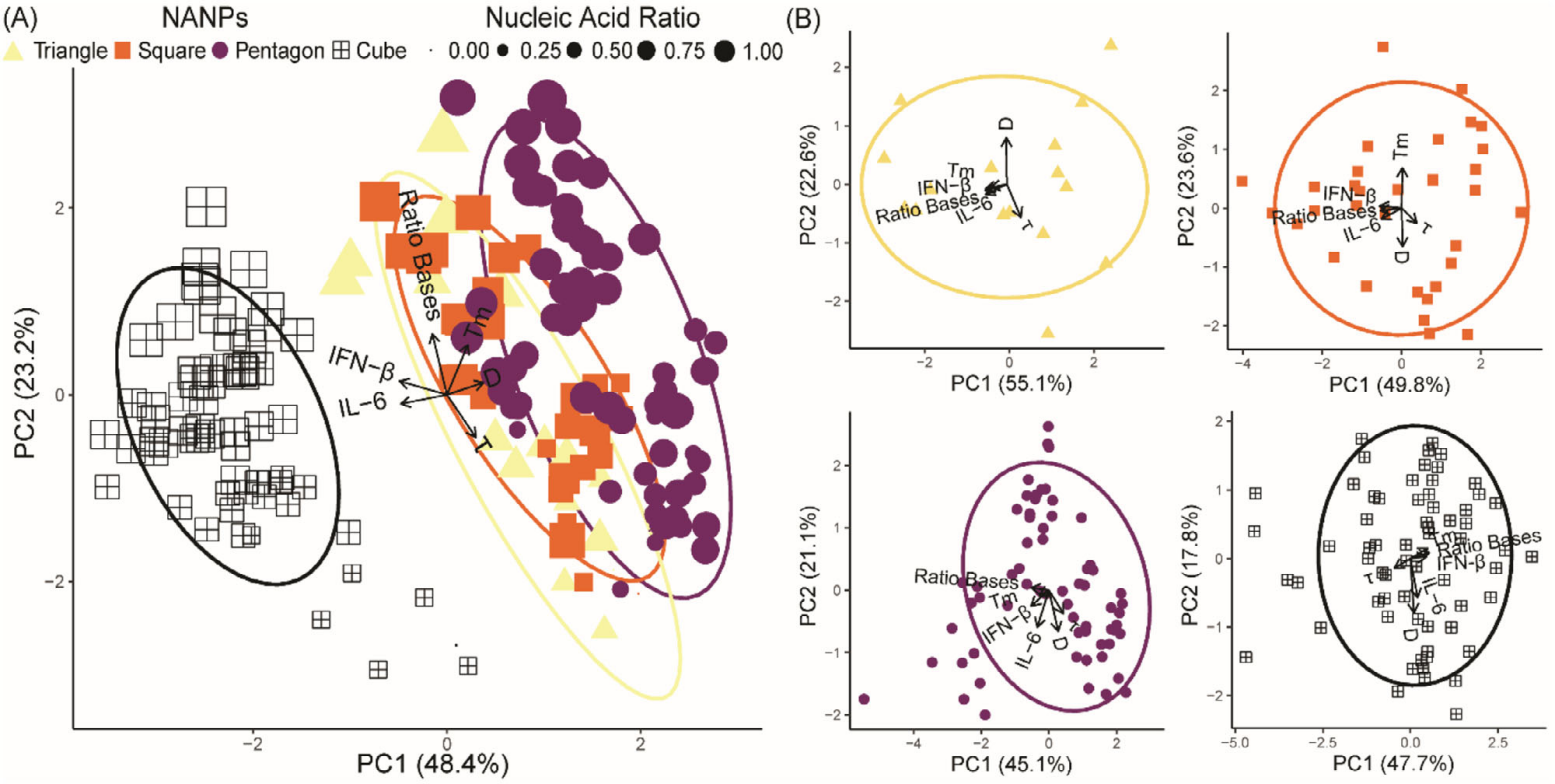
PCA plots of NANPs. Empirical determination of IFN‐β, IL‐6, FBS stability (τ), and melting temperatures (T_m_) expressed as the mean for a minimum of three independent replicates was recorded to identify differential immune responses and general stability of the NANPs in vitro. All ellipses represent the 85% confidence interval of the respective shape. The length of the arrows determines how strongly each factor influences the distribution of the first two axes of the PCA, and the direction determines its trend and correlation to the other factors. A) PCA plot of all NANP conformations. The shape of each point is determined by the conformation of the NANP, and the size of the point is the proportion of strands that are composed of RNA, compared to the entire structure. B) Individual PCA plots of each NANP architecture (triangles, squares, pentagons, and cubes).

**Figure 4:**
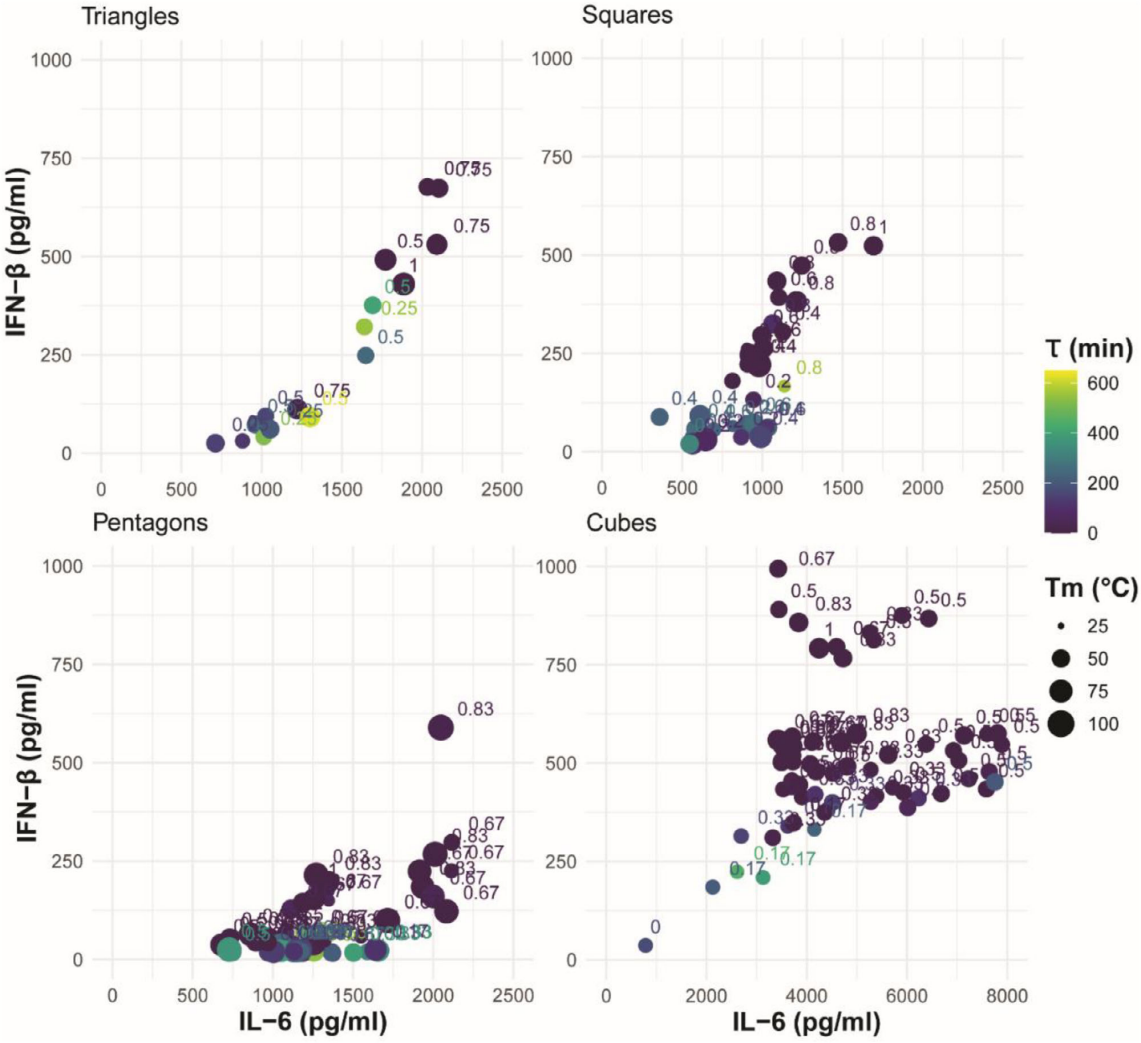
Scatter plots of the physicochemical and immunostimulatory properties of 2D and 3D NANPs. Scatter plots represent the mean protein production of IL‐6 and IFN‐β by hµglia cells in response to transfection with 5 nm NANPs for three independent replicates. The size of each point represents the mean melting temperature for three independent replicates for each NANP, while the color of the point represents NANPs relative mean stability in serum for three independent replicates.

Here, we defined the production of inflammatory cytokine, IL‐6, and type I interferon, IFN‐β by human microglia following exposure to the comprehensive panel of 176 NANPs as indicators of immune stimulation (Figure S10 and S11, Supporting Information). **Figure** **3** demonstrates microglia production of IL‐6 and IFN‐β in relation to the physicochemical properties for the polygonal and cubical NANPs. Figure 3A combines all structural configurations, and the first two components illustrate 71.6% of the variance in the data. Notably, principal component analysis (PCA) demonstrates that globular 3D NANPs form a distinct group from the planar 2D triangle, square, and pentagon NANPs, consistent with our previous observations that dimensionality is a fundamental predictor of NANP physicochemical and immunostimulatory properties. The PCA plot highlights the inverse relationship between the ratio of RNA bases to total bases and serum stability, which is indicative of the susceptibility of RNA to nuclease activity. Additionally, we observe that 3D NANPs stimulate production of IFN‐β ranging from 750–1000 pg mL^−1^ and IL‐6 ranging from 3000–8000 pg mL^−1^. While 2D NANPs trigger production of IFN‐β and IL‐6 by microglia, it remains below 750 and 2500 pg mL^−1^ for IFN‐β and IL‐6, respectively (**Figure** **4**). The individual PCA plots for each shape shown in Figure 3B demonstrate unique relationships among the measured physicochemical properties. Specifically, triangles have a strong positive correlation for all physicochemical properties except for tau and diameter, which are inversely correlated. Squares have an inverse relationship with diameter and melting temperature, with a strong positive correlation with IL‐6, the proportion of the RNA bases, and IFN‐β. This is in contrast to pentagons, which generally have positively correlated physicochemical properties. Interestingly, the cube configuration results in a negative correlation in the serum stability against melting temperature, ratio of RNA, and IFN‐β, while the diameter and IL‐6 responses are positively correlated. Collectively, these data support distinct relationships between physicochemical properties for 2D and 3D NANP configurations.

Consistent with our previous findings, as the number of RNA strands increases, there is a corresponding increase in the production of immune mediators by microglia, indicating nucleic acid composition is a fundamental predictor of NANP immunostimulatory properties. This trend is observed in both our planar and globular NANPs (Figure 4). Importantly, all NANPs composed of predominantly or exclusively DNA stimulate minimal to no production of IFN‐β and IL‐6.

Finally, the individual PCA plots demonstrate physicochemical properties that are correlated with NANP immunostimulatory properties. For triangle NANPs, there is a strong positive correlation between IFN‐β and IL‐6 production, the ratio of RNA bases to total bases, and melting temperature. Interestingly, as the total number of nucleic acid strands increases in the 2D NANPs we see variation in this positive correlation between immune stimulation, RNA bases, and melting temperature. For example, we observe that the immune mediator production of IL‐6 and IFN‐β in response to square NANPs is positively correlated only with the ratio of RNA bases. Additionally, only the stimulation of IFN‐β production by microglia exposed to pentagon NANPs remains positively correlated with melting temperature. Similarly, we observed that only IFN‐β production by microglia in response to 3D cube NANPs is correlated with melting temperature and the ratio of RNA bases. Collectively, the physicochemical and immunostimulatory characterization of this panel is extensive and consistent with the published fundamental predictors of NANP immune stimulation and is therefore an ideal data set for predictive modeling.

### 2.3 Sequence‐Based QSAR Modeling Predicts NANP‐Induced Immune Responses

**Figure 5:**
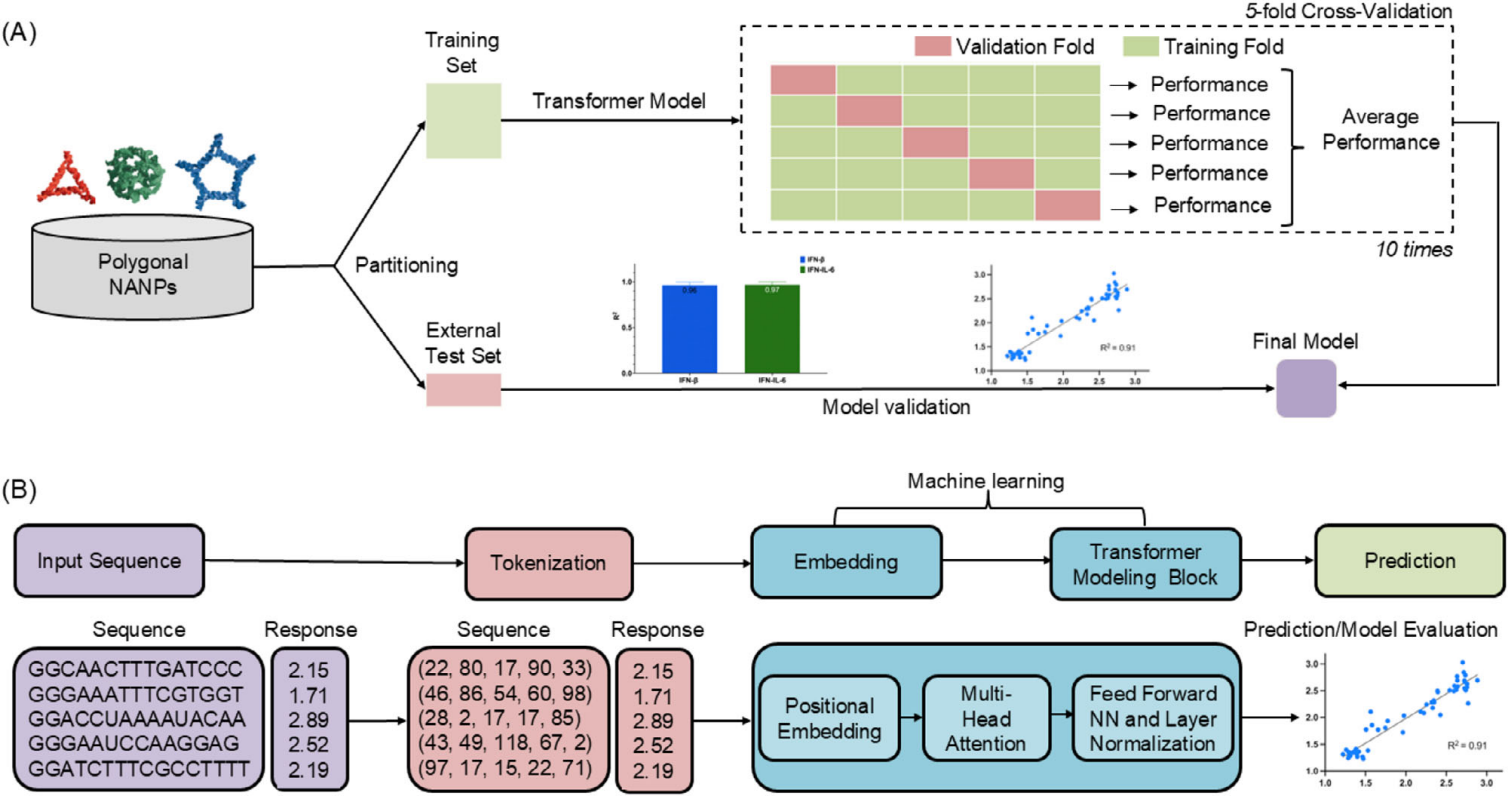
Overview of the transformer‐based modeling workflow for predicting NANP‐induced cytokine responses. A) Schematic of the model training and evaluation process. Polygonal nucleic acid nanoparticles (NANPs) are partitioned into training and external test sets. A transformer model is trained using 5‐fold cross‐validation repeated 10 times, with performance averaged across iterations and validated on an external test set. B) Sequence‐based modeling pipeline. NANP sequences are first tokenized and embedded, then passed through a transformer architecture to generate cytokine response predictions.

**Figure 6:**
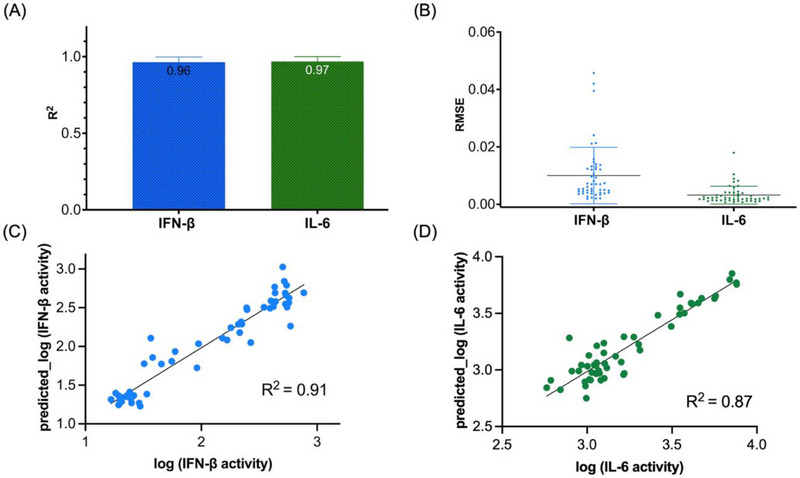
Average model performance on the training set showing (A) R^2^ and (B) RMSE for IFN‐β and IL‐6 over 5‐fold cross‐validation, repeated 10 times (*n* = 123). Error bars indicate the standard deviation across 50 iterations. Panels (C,D) show scatter plots of observed versus predicted log‐transformed values for IFN‐β and IL‐6 on the external test set, using the best model selected from training (*n* = 53). The test set R^2^ values are 0.91 and 0.87, respectively.

QSAR modeling was employed to predict immune responses based on NANP strand sequences. We selected a transformer‐based architecture (Transformer_M1, see Materials and Methods) for its superior ability to capture sequence‐dependent interactions (**Figure** **5**).^[^ ^11^ ^]^ The model demonstrated strong predictive performance for both IFN‐β and IL‐6 production by human microglial cells based. On the training set (70%), the model achieved an average R^2^ of 0.96 ± 0.03 and RMSE of 0.01 ± 0.01 for IFN‐β, and an R^2^ of 0.97 ± 0.03 with RMSE of 0.003 ± 0.003 for IL‐6, indicating high accuracy. On the external test set (30%), the best‐performing model from cross‐validation maintained generalizability with R^2^ values of 0.91 for IFN‐β and 0.85 for IL‐6, and the corresponding RMSE values of 0.17 and 0.12, respectively (**Figure** **6**). These results validate the transformer model's robustness in predicting immune responses across structurally diverse NANP sequences.

For context, we also evaluated a descriptor‐based Random Forest, an LSTM, and a transformer without augmentation under the identical split and metrics. A concise summary is in Figure S12 and S13 and TableS5A, B (Supporting Information). Differences in model performance (R^2^ and RMSE) were assessed with the Friedman nonparametric test; results are reported in TableS6 (Supporting Information). On the external test, Transformer_M1 again performed best on both endpoints (scatter overlays in Figure S14 (Supporting Information), full comparator metrics Table S7A–B, Supporting Information), and therefore we used it for all sequence‐only predictions in the main text.

## 3 Discussion

ML and deep learning approaches are increasingly transforming the landscape of biomedical research, particularly in modeling complex relationships between biomolecular sequences and functional outcomes. While these methods have shown remarkable success in genomics, drug discovery, and QSAR modeling, their application to NANP technologies remains underexplored. NANPs are highly programmable and structurally diverse; as such, their experimental characterization, particularly immunological profiling, is time‐consuming, requiring multi‐step synthesis, assembly validation, and cytokine analysis. These challenges necessitate the development of predictive frameworks capable of guiding rational design from molecular sequence data while reducing experimental burden.

In this study, we present a transformer‐based deep learning framework to predict key immune responses – IFN‐β and IL‐6, induced by representative NANPs in human microglial cells. The model was trained on a diverse panel of 176 NANPs and evaluated through repeated cross‐validation and external testing. Unlike traditional QSAR approaches such as Random Forest (RF), which rely on pre‐defined features like physicochemical descriptors and often fail to capture higher‐order dependencies, our architecture learns directly from nucleotide sequences.^[^ ^45^, ^46^ ^]^ By leveraging self‐attention mechanisms, the transformer model captures positional and contextual relationships across multi‐stranded constructs, enabling it to model sequence‐dependent interactions with high fidelity.^[^ ^47^ ^]^ This approach avoids the limitations of manual feature engineering and provides a scalable, generalizable framework for sequence‐driven immunogenicity prediction in structurally diverse NANPs.

A key aspect of this framework is the use of systematic strand permutation to augment the training data. As the physical connectivity between strands is not explicitly represented in sequence‐based inputs, we adopted the strand permutation strategy introduced in our prior AI‐cell study to expose the model to a range of possible strand orderings for each NANP. In this work, Transformer_M1 trained on the augmented sequences learned features that are effectively topologically invariant, enhancing its robustness and generalization across nanoparticle configurations with varying architectures, while maintaining high predictive accuracy for both IFN‐β and IL‐6 responses.

The results demonstrate that the transformer‐based model effectively captures biologically relevant immune responses directly from NANP sequences. It accurately predicted both IFN‐β and IL‐6 production in human microglial cells, confirming its ability to generalize beyond the training data without relying on structural or physicochemical features. This suggests that immunogenic patterns may be encoded in sequence‐level composition and context, and that attention‐based models can learn these signals even in structurally complex, multistranded systems. Importantly, the use of microglia, a CNS‐resident innate immune cell type extends the modeling framework into a neuroimmune context with direct translational relevance. Microglia possess distinct nucleic acid sensing mechanisms and play critical roles in neuroinflammation, positioning them as a physiologically meaningful model for evaluating NANP‐induced cytokine responses. By enabling *in silico* modeling and profiling of these responses, our NAM contributes to efforts in designing immune‐engineered TNAs tailored for CNS delivery, while also supporting broader goals in improving the predictive relevance of preclinical testing and reducing experimental burden during early‐stage therapeutic development.

This sequence‐based framework eliminates the need for labor‐intensive feature engineering and physicochemical descriptor calculations while maintaining strong predictive performance. Its ability to operate directly on nucleic acid sequences makes it particularly advantageous for large‐scale datasets and time‐sensitive applications, such as personalized immunotherapy design or early‐stage screening. By streamlining the modeling pipeline, this approach offers a scalable solution for rational NANP development, even in settings where structural information is incomplete or high‐throughput experimental profiling is not feasible. Future extensions of this work could incorporate additional immune endpoints or explore other nanoparticle architectures to further expand its translational scope in nanomedicine and immunotherapy.

To facilitate broad adoption, we have deployed the top‐performing models via an updated version of the AI‐cell web platform (https://aicell.ncats.io). This user‐friendly interface enables real‐time prediction of cytokine responses from user‐supplied NANP sequences, supporting hypothesis generation, design iteration, and preclinical risk assessment. Taken together, this study advances sequence‐based QSAR modeling by demonstrating that attention‐based deep learning architectures can extract biologically meaningful immune signatures directly from nucleic acid sequences. Through the integration of curated experimental data, principled model design, and public deployment, we established a generalizable framework for applying AI to the immune profiling and rational engineering of therapeutic nucleic acid nanostructures.

## 4 Experimental Section

### NANPs Assembly

All sequences used in this study are listed in Table S1 (Supporting Information). DNA strands for the assembly of NANPs and templates for RNA strands transcription were purchased from Integrated DNA Technologies (Coralville, IA, USA). DNA templates for RNA strands were PCR‐amplified using MyTaq™ Mix (Bioline, London, UK), and the resulting products were purified with the DNA Clean and Concentrator™ kit (Zymo Research, Irvine, CA, USA). In vitro run‐off transcription (IVT) was performed using T7 RNA polymerase under the following conditions: 80 mm HEPES‐KOH (pH 7.5), 2.5 mm spermidine, 50 mm DTT, 25 mm MgCl_2_, and 5 mm rNTPs. IVTs were incubated at 37 °C for 3.5 h, followed by treatment with RQ1 RNase‐free DNase (Promega, Madison, WI, USA) to stop the reaction and remove DNA templates. Transcribed RNA was purified using 15% denaturing PAGE containing 8 M urea. RNA bands were visualized under short‐wavelength UV light, excised, and eluted overnight in a crush‐and‐soak buffer (300 mm NaCl, 89 mm Tris‐borate, pH 8.2, 2 mm EDTA). RNA was precipitated with two volumes of 100% ethanol at −20 °C for 3 h, pelleted by centrifugation at 14000 × g for 30 min, and washed twice with 90% ethanol for 10 min each. Pellets were vacuum‐dried and resuspended in endotoxin‐free (ET‐free) water. All NANPs were individually assembled in a one‐pot reaction by combining purified monomers at equimolar concentrations in ET‐free water. For cube NANPs, the mixtures of six monomers at equimolar concentrations were heated to 95 °C, snap‐cooled to 45 °C, and incubated for 2 min before the addition of assembly buffer (89 mm Tris‐borate, pH 8.2, 2 mm MgCl_2_, 50 mm KCl). Assembly was completed by incubating the mixtures at 45 °C for an additional 20 min. Fully assembled NANPs were stored at 4 °C for subsequent experiments. All polygon NANPs were assembled from equimolar mixtures of the corresponding RNA, DNA, or a combination of RNA and DNA strands heated to 95 °C and then slowly (1 h) cooled to 4 °C in assembly buffer.

### Atomic Force Microscopy (AFM)

Eight representative NANPs (Figure S1, Supporting Information) were deposited on APS‐modified mica, incubated for ≈2 min, and air‐dried, as detailed in our previously established protocols.^[^ ^48^ ^]^ AFM imaging was carried out using a MultiMode AFM Nanoscope IV system (Bruker Instruments, CA), in tapping mode. The images were collected with a 1.5 Hz scanning rate using a TESPA‐300 probe from Bruker with a resonance frequency of 320 kHz and spring constant of ≈40 N m^−1^. Images were processed by the FemtoScan Online software package (Advanced Technologies Center).

### NANPs Hydrodynamic Diameter Measurements

Assembled NANPs were prepared at a concentration of 1 µm in a final volume of 150 µL in assembly buffer. Samples were briefly centrifuged at 14000 x g for 5 min. The 100 µL volume was then transferred to a micro cuvette (Starna Cells) for DLS analysis. DLS measurements were performed at 25 °C using a Zetasizer Nano‐ZS (Malvern Instruments Ltd., Malvern Panalytical Ltd., Malvern, UK) with backscattered light detection at 175°. Each sample was scanned 15 times, and the procedure was repeated in triplicate.

### UV‐Melting Temperature

Assembled NANPs were prepared at a concentration of 0.2 µm in a final volume of 100 µL. Samples were degassed for 5 min using a CentriVap (Labconco) to remove any air bubbles. The resulting ≈100 µL of sample was transferred to a UV‐melting cell (Starna Cells micro cuvette) equipped with a PTFE stopper to prevent evaporation. For UV‐melting analysis, absorbance measurements were collected from 20 to 100 °C with a heating ramp of 1 °C per minute using an Agilent 8453 spectrophotometer coupled with an Agilent 89 090 Peltier Temperature Controller. Cube‐shaped nanoparticles were analyzed using a Shimadzu UV‐2600i UV–Vis spectrophotometer equipped with a Thermal Melt System TMSPC‐8. Each sample was placed in an 8 series Micro Cell with 1 cm path length. Teflon cell stoppers were used with each cuvette to prevent sample evaporation throughout the experiment. Temperature control was maintained by a water bath to dissipate heat from the TMSPC‐8 thermal block. The melting temperature (Tm) was calculated using the Boltzmann sigmoidal function to fit the melting data in OriginPro (OriginLab) software. This function models the sigmoidal shape of the melting curve as a function of temperature according to the equation:

(1) $$F\left(T\right)=A_{1}+\frac{A_{2}-A_{1}}{1-e^{\frac{T-T_{m}}{w}}}$$

where *T* was the temperature, *T_m_* was the melting temperature, *w* represents the slope of the curve at the *T_m_*, and *A1*​ and *A2* were the lower and upper baseline values (asymptotes) of the curve, respectively and *A2−A1*​ was the amplitude of the curve. This equation assumes cooperative melting behavior and a two‐state model (binary), enabling a sigmoidal representation of the fraction of hybridized nucleic acid strands as a function of temperature.

### Serum Stability Assay

Assembled NANPs (1 µm) were incubated in a 20% (v v^−1^) fetal bovine serum solution at 37 °C over various time intervals, ranging from 1 min to 720 min (12 h). At each designated time point, 10 µL aliquots were collected and immediately placed on dry‐ice to halt further degradation. Samples were then mixed with loading dye (product ID) and subjected to 3% agarose gel electrophoresis under non‐denaturing conditions to separate and visualize the remaining intact NANP fractions. Gels were made with Ethidium Bromide and imaged under Gel Doc (BioRad) and Typhoon 5 (Cytiva) Biomolecular imager to assess the stability profile of the NANPs across the time course.

### Cell Maintenance of Human Microglia (Hµglia)

The immortalized primary hµglia cells employed in these studies were a generous gift from Dr. Jonathan Karn (Case Western Reserve University). Primary human cells were transformed with lentiviral vectors expressing SV40 T antigen and hTERT, followed by characterization of cellular morphology, migratory and phagocytic activity, surface markers, and RNA expression profile.^[^ ^49^ ^]^ Cells were maintained in Dulbecco's modified Eagle's medium supplemented with 5% FBS and 1% penicillin/streptomycin (100 U mL^−1^ – 100 µg mL^−1^).

### NANP Delivery to Hµglia

NANPs were delivered to hµglia cells using Lipofectamine 2000 (L2K) according to published protocols^[^ ^6^, ^7^, ^8^, ^24^ ^]^ and the manufacturer's guidelines. Briefly, NANPs were incubated for 30 min with L2K prior to transfection of hµglia with NANPs (5 nM, final concentration) for 4 h in DMEM supplemented with 5% FBS. At 4 h post transfection the cell culture media were changed to media supplemented with 100 U mL^−1^ penicillin‐100 µg mL^−1^ streptomycin, and cell supernatants were collected for analysis at the indicated time points.

### Enzyme‐Linked Immunosorbent Assay (ELISA)

Specific capture ELISA was performed to quantify human IL‐6 and IFN‐β production according to our published studies.^[^ ^6^, ^7^, ^9^, ^50^ ^]^ Briefly, a rat anti‐human IL‐6 capture antibody (BD Pharmingen, cat# 554 543, Clone Mq2‐13A5) and a biotinylated rat anti‐human IL‐6 detection antibody (BD Pharmingen, cat# 554 546, Clone MQ2‐39C3) were used for IL‐6 specific capture ELISAs. To detect IFN‐β, a polyclonal rabbit anti‐human IFN‐β capture antibody (Abcam, cat# ab186669) and a biotinylated polyclonal rabbit anti‐human IFN‐β detection antibody (Abcam, cat# ab84258) were used in specific capture ELISAs. Prior to the addition of tetramethylbenzidine substrate, Streptavidin–horseradish peroxidase (HRP) (BD Biosciences) was added to detect bound antibody. The reaction was stopped using H_2_SO_4_ and the absorbance was measured at 450 nm. Recombinant cytokines for IL‐6 (BD Pharmingen) or IFN‐β (Abcam) were employed to generate standard curves and the concentration of IL‐6 and IFN‐β in cell supernatants was determined by extrapolation of absorbance to the standard curve.

### Dataset for Modeling

In this study, sequences of 176 nucleic acid nanoparticles (NANPs) were used to construct computational models to predict immune responses, with interferon‐beta (IFN‐β) and interleukin‐6 (IL‐6) production in human microglial cells as the target variables. The immune response data associated with these cytokines are detailed in Table S2 (Supporting Information). For modeling, IFN‐β and IL‐6 target values were log10‐transformed.

The dataset was split into a training set (70%) and an external test set (30%) to evaluate model performance comprehensively. On the training set, a five‐fold cross‐validation repeated 10 times was conducted to ensure robustness. This process allowed for the selection of the best‐performing model based on RMSE and R^2^, which was subsequently evaluated on the external test set to assess its predictive accuracy on unseen data. The train‐test split was essential to prevent overfitting and to gauge how well the model generalizes to new, unseen data.^[^ ^1^, ^2^, ^51^ ^]^ By reserving a portion of the data for external testing, the model's performance could be understood better in real‐world applications, and its reliability^[^ ^52^ ^]^ in predicting immune responses for new NANP sequences.

### Model Development

Building on the transformer architecture presented by Chandler et al.,^[^ ^10^ ^]^ a similar sequence‐based transformer model was implemented to predict the immune responses for IFN‐β and IL‐6. The choice to use transformer models was driven by their ability to handle complex relationships in sequence data through self‐attention mechanisms.^[^ ^45^, ^53^ ^]^ Unlike traditional machine learning methods such as RF, transformers have shown superior performance in capturing context dependencies and relationships within the sequence,^[^ ^54^, ^55^ ^]^ which were crucial for predicting immune responses triggered by nucleic acid sequences. Along with the transformer, two models were compared for context: a descriptor‐based Random Forest and an LSTM. Full configurations and results are provided in the Supporting Information.

### Tokenization and Data Augmentation

The first step in sequence‐based modeling was tokenization. A k‐mer strategy (k = 3) was used, breaking down sequences into overlapping triplets (e.g., AGT, GTC). Since the exact arrangement of strands within NANPs was uncertain for modeling, data augmentation was used to generate all possible strand permutations. This approach significantly expanded the dataset, allowing the model to capture different potential strand configurations and interactions, ensuring robust and unbiased predictions.^[^ ^13^ ^]^ It was referred to the models generated using this approach as Transformer_M1, with additional details provided in the Supporting Information.

### Transformer Model Architecture

The transformer model included an embedding layer, transformer blocks with multi‐head self‐attention, and a final regression output layer. The embedding layer converted tokenized sequences into dense numerical vectors for the model to learn contextual information. Transformer blocks, consisting of self‐attention and feed‐forward networks, captured relationships within sequences.^[^ ^56^ ^]^ A global average pooling layer selected relevant features before the final output layer. The model was trained using the Adam optimizer and mean absolute error (MAE) loss function. The network architecture and training parameters are provided in TablesS3 (Supporting Information).

### Cross‐Validation and Performance Metrics

To ensure robustness, the model was evaluated using a five‐fold cross‐validation strategy, repeated 10 times.^[^ ^13^, ^57^ ^]^ The performance of the model was assessed using two key metrics:

(2) $$R^{2}=1-\frac{\sum_{i}^{n}({\left({\overset{⌢}{Y}}_{t}-Y_{i}\right)}^{2}}{\sum_{i}^{n}({\left(Y_{i}-\bar{Y}\right)}^{2}}$$

(3) $$\mathrm{RMSE}=\sqrt{\frac{1}{n}\sum_{i}^{n}{\left({\overset{⌢}{Y}}_{t}-Y_{i}\right)}^{2}}$$

Y_i_(cap) was the predicted value for each particular sequence; *Y_i_* was the observed value for each particular sequence; *Y_i_(bar)* was the mean activity value from all the sequences; *n* was the number of sequences. *R^2^* (coefficient of determination): Measures the proportion of variance explained by the model; *RMSE* (root mean squared error): Reflects the average magnitude of the prediction errors. The model's predictions for IFN‐β and IL‐6 cytokine responses were compared against experimentally observed values, and the mean *R^2^* and *RMSE* were reported over all folds.

### RStudio Analysis

The QSAR table was imported into RStudio, with R version 4.4.0.^[^ ^58^ ^]^ The normalized values of the data were input into the function prcomp from the stats package.^[^ ^58^ ^]^ Next, the loadings calculations were generated using the factoextra version 1.0.7.^[^ ^59^ ^]^ The final biplot and scatterplot graphs were plotted with the package ggplot2 version 3.5.1,^[^ ^60^ ^]^ and the color schemes were adapted from the viridis package version 0.6.5.^[^ ^61^ ^]^ The four individual shape PCA's were aggregated using the plotgrid command from the cowplot package version 1.1.3.^[^ ^61^ ^]^ Next, a heatmap was generated using raw data as input into the ComplexHeatmap package version 2.20.0.^[^ ^62^, ^63^ ^]^

### Statistical Analysis

Analyses were performed in Python 3.9.13 (numpy, scipy, scikit‐learn, statsmodels, pandas, matplotlib) with TensorFlow 2.0.0 for model training, and in GraphPad Prism 9.0.0 (Windows; GraphPad Software, CA). For model benchmarking, differences in performance metrics (R^2^, RMSE) across methods were assessed using the Friedman nonparametric test applied to the repeated five‐fold CV results; where relevant, pairwise contrasts were examined on the same ranking framework. Unless stated otherwise, tests were two‐sided with α = 0.05. Data are presented as mean ± SD, with the sample size (n) reported for each analysis. Targets (IFN‐β, IL‐6) were modeled on the log10 scale; metrics were computed on that scale unless noted. Compute environment and wall‐time details for transformer training are summarized in Table S4 (Supporting Information).

## Conflict of Interest

The authors declare no conflict of interest.

## Author Contributions

M.B.J. and S.J. contributed equally to this work. M.B.J. did the investigation, formal analysis, and Writing‐ original draft. S.J. did data analysis, computational modeling, model deployment via AI‐cell web platform, and Writing – original draft. J.M.S. did the investigation and formal analysis. Q.K. did formal analysis, Writing‐ original draft. E.D. did investigation, formal analysis, and Writing‐ original draft. D.M. did the investigation, formal analysis. K.P. did the investigation, formal analysis. H.H. did the investigation, formal analysis. A.T. did the investigation, formal analysis. J.H. did the investigation, formal analysis. E.K. did formal analysis, writing‐ original draft, and funding acquisition. A.V.Z. did conceptualization, formal analysis, and Writing‐ original draft. K.A.A. did conceptualization, formal analysis, Writing‐ original draft, funding acquisition

## Supporting information



Supporting Information

Supplemental Table 1

## Data Availability

The data that support the findings of this study are available from the corresponding author upon reasonable request.
