# Supplementary material for: From Sequence to Response: AI-Guided Prediction of Nucleic Acid Nanoparticles Immune Recognitions
Source: Small. Author manuscript; Available in PMC 2025 Dec 18. (PMC12710182; doi:10.1002/smll.202509459)
Supplement: SI [file NIHMS2123988-supplement-SI.docx]

**Supporting Information for**

**From Sequence to Response: AI-Guided Prediction of Nucleic Acid Nanoparticles Immune Recognitions**

M. Brittany Johnson^1^*^#^, Sankalp Jain^2#^, Jessica McMillan Shea^3^, Quinton Krueger^1,4^, Erwin Doe^5^, Daniel Miller^5^, Katelynn Pranger^5^, Hannah Hayth^5^, Sable Thornburgh^5^, Emil Khisamutdinov^5^*, Alexey V. Zakharov^2^*, and Kirill A. Afonin^3^*

^1^Department of Biological Sciences, University of North Carolina at Charlotte, 9201 University City Boulevard, Charlotte, NC, 28223, USA.

^2^National Center for Advancing Translational Sciences, National Institutes of Health, Rockville, MD, 20850, USA.

^3^Nanoscale Science Program, Department of Chemistry, University of North Carolina at Charlotte, Charlotte, NC, 28223, USA.

^4^Computational Intelligence for Predicting Health and Environmental Risks (CIPHER), University of North Carolina at Charlotte, Charlotte, NC, USA 28223

^5^Department of Chemistry, Ball State University, Muncie, IN, 47306, USA.

# These authors contributed equally to this project.

***** correspondence to mjohn398@charlotte.edu (MBJ), [kemil@bsu.edu](mailto:kemil@bsu.edu) (EK), alexey.zakharov@nih.gov (AVZ), kafonin@charlotte.edu (KAA).

**Supporting Methods**

**Nanoparticle Sequence Tokenization:** Tokenization is the first step for processing input sequence data in a sequence-to-sequence model. It changes the raw text input into a sequence of discrete units, called tokens. For the nanoparticle sequences, we used the K-mer representation with K=3. This method loosely encodes triplets, similar to codons, by generating all possible combinations of 3-mers from the individual nucleotide bases (A, T, G, C, U). This K-mer method is helpful because it includes rich, local contextual information for each base by looking at its immediate neighbors. This process produced a total of 125 unique 3-mer tokens, which together formed the vocabulary. Each input sequence in the training dataset was then tokenized and sent through an embedding layer. This layer mapped these 3-mer tokens to their respective vector representations for model processing

**Data Augmentation by Generating Nanoparticle Combinations:** The composition of NANPs defines their immune response. When we try to apply this biological activity, like IFN induction, to a sequence-based learning model, knowing the exact order of individual strands required to trigger a specific response is uncertain. To solve this issue, we created all possible sequence arrangements for each nanoparticle by rearranging its strands. For a NANP made of n strands, this leads to n! unique combinations. We assigned the biological activity measured, such as IFN activity, from the original NANP to every combination we generated. This approach greatly increased the training dataset for developing the final models, which we call Transformer_M1.

**Cross-Validation Strategy:** To avoid information leakage during the 5-fold external cross-validation, we made sure all combinations generated from a single NANP were grouped together. Therefore, when we split the augmented dataset, all n! combinations of a specific NANP were either entirely in the training set or entirely in the validation set. This method ensured there was no overlap of parent NANPs between the training and validation splits. We then calculated model performance metrics based on the average prediction values across all combinations for each original NANP.

**SUPPLEMENTAL FIGURES**

**
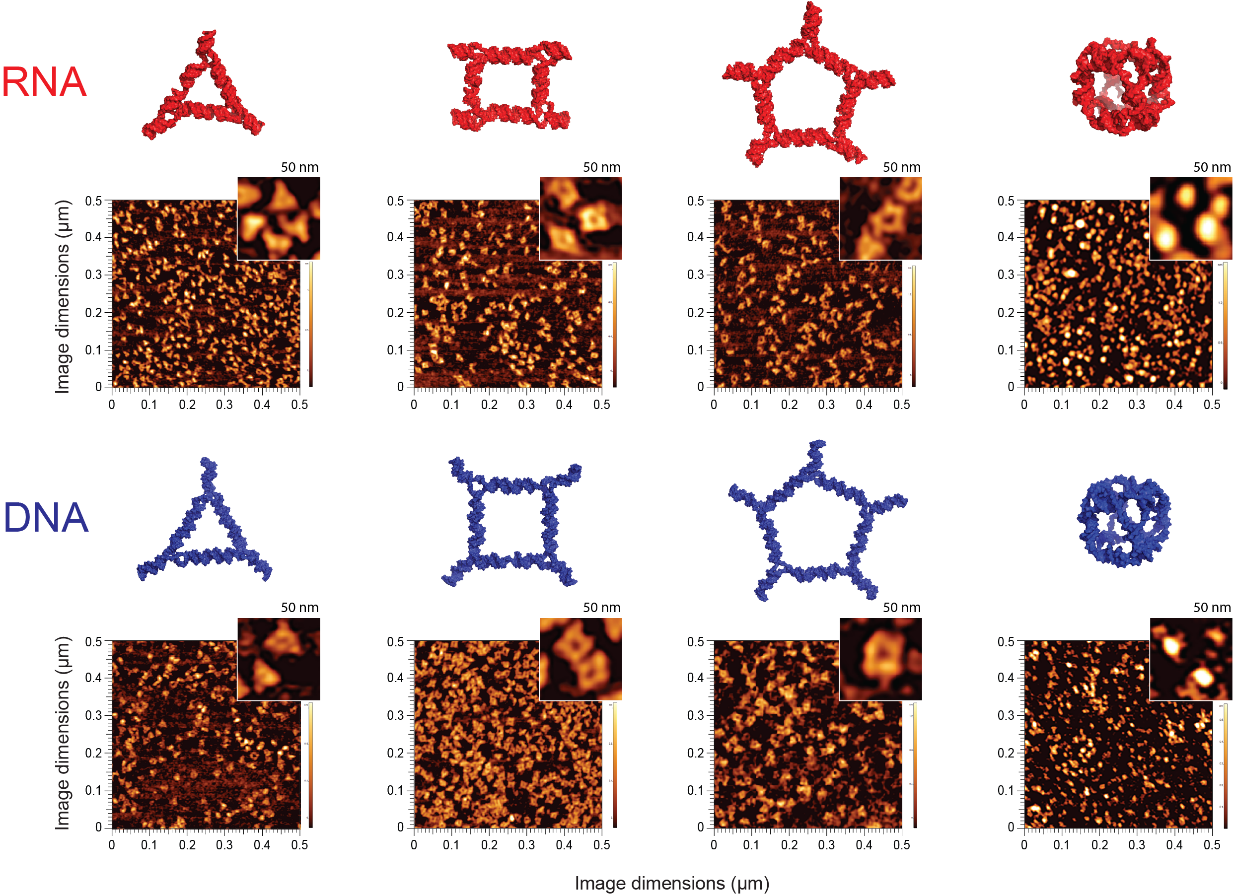
**

**Supplemental Figure S1.** Representative AFM images of four main architectures of nucleic acid nanoparticles (NANPs) composed of either RNA or DNA tested in this study. The images confirm the correct formation of the intended nanostructures.

**
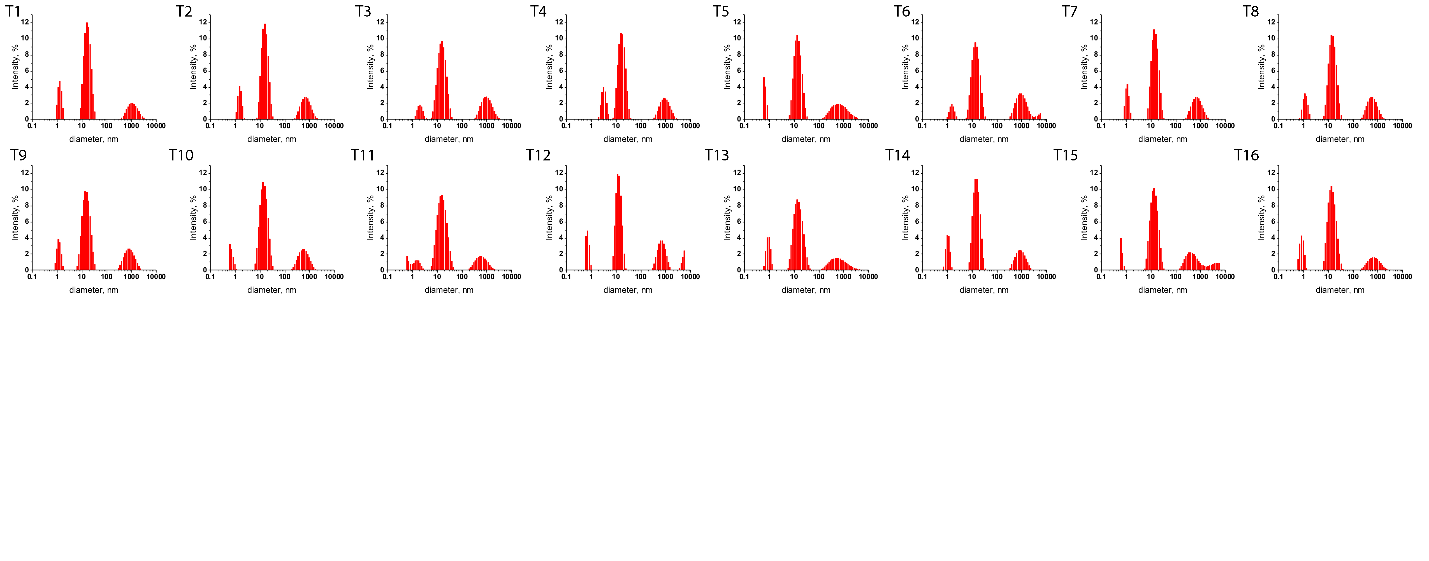
**

**
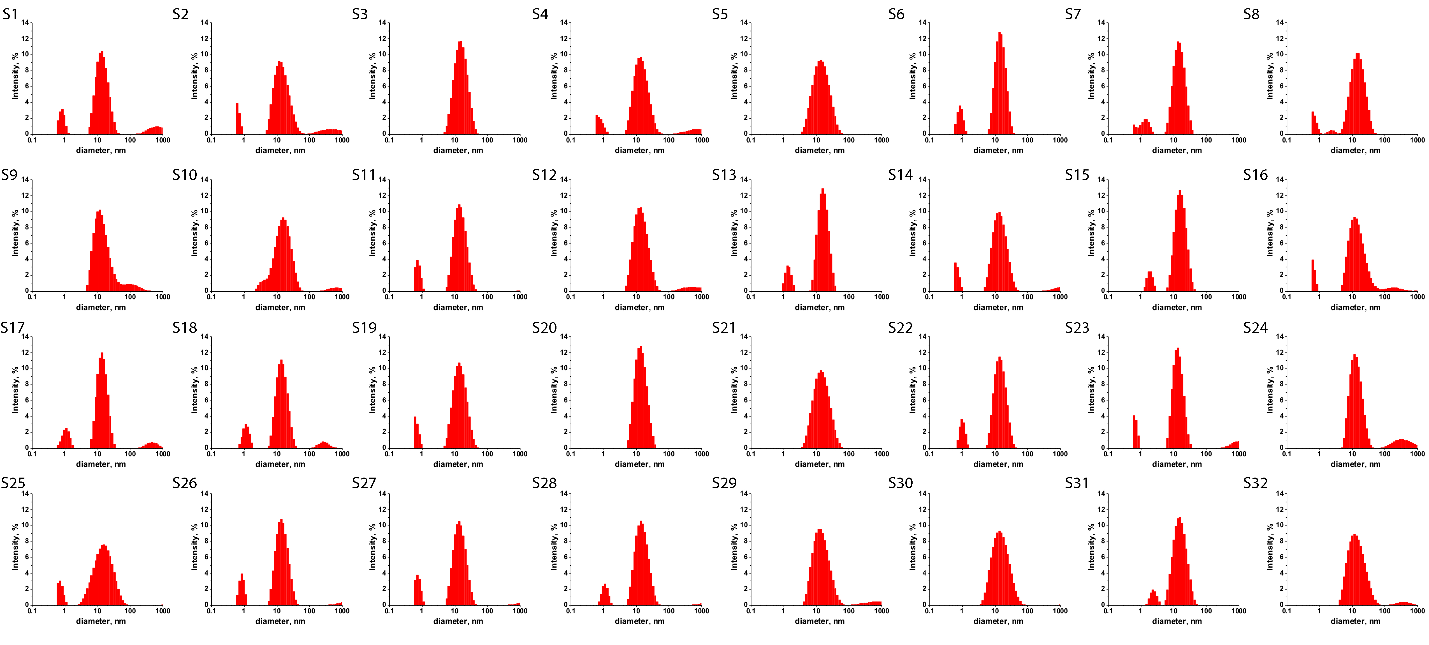
**

**Supplemental Figure S2.** Representative DLS spectra of nucleic acid nanoparticles (NANPs). DLS analysis was performed on a series of assembled polygonal structures, including a set of 16 triangular (T1–T16) and 32 square (S1–S32), and 64 pentagonal (P1–P64) NANPs. Size distributions from the DLS measurements indicate the hydrodynamic diameters of each structure. The polydispersity indices for the triangular and square nanoparticles were within the moderate range of 0.3–0.6.


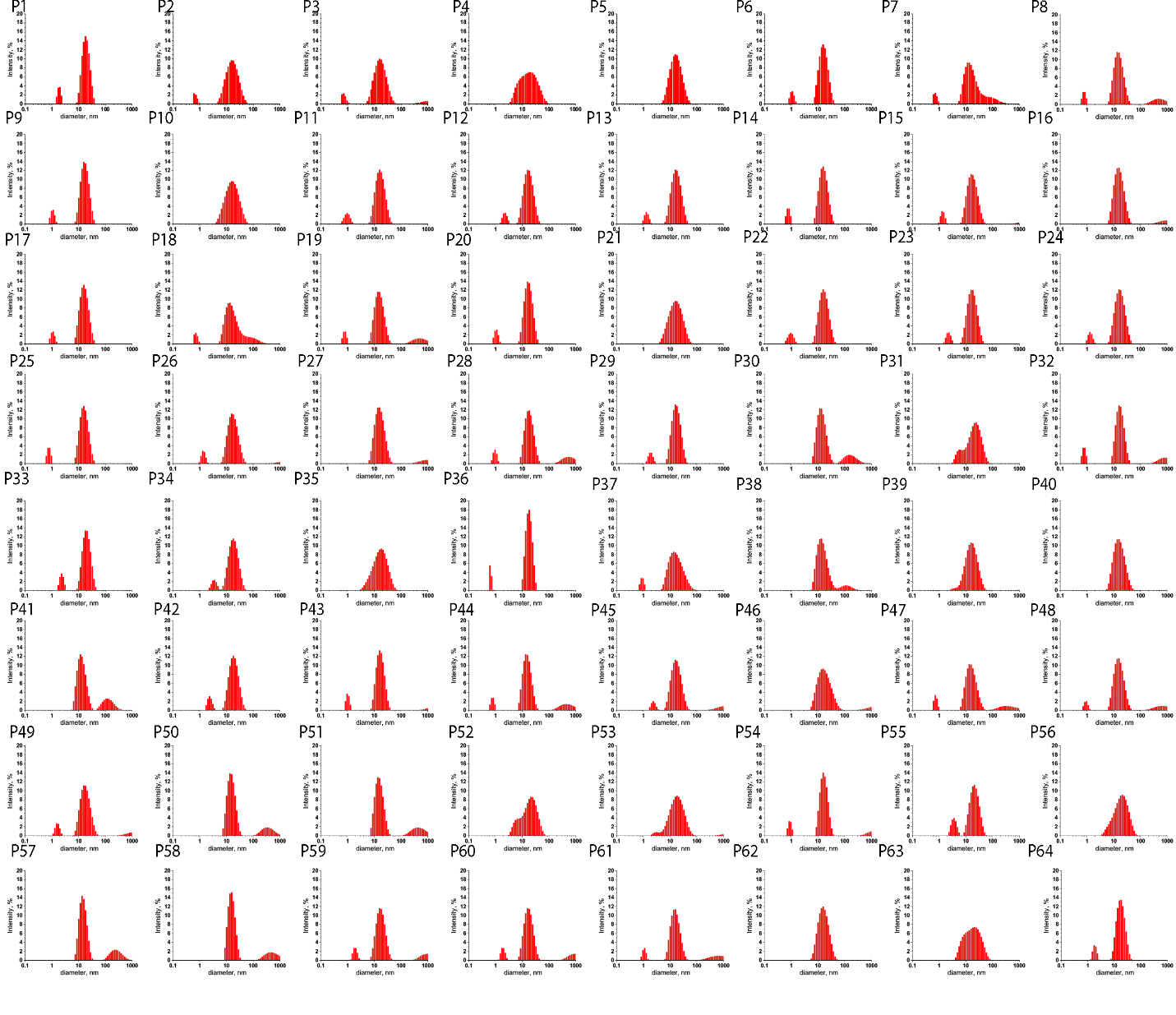


**Supplemental Figure S3.** Representative DLS spectra of nucleic acid nanoparticles (NANPs). DLS analysis was performed on a series of 64 pentagonal (P1–P64) NANPs. Size distributions from the DLS measurements indicate the hydrodynamic diameters of each structure. The polydispersity indices for the pentagonal nanoparticles were within the moderate range of 0.3–0.6.


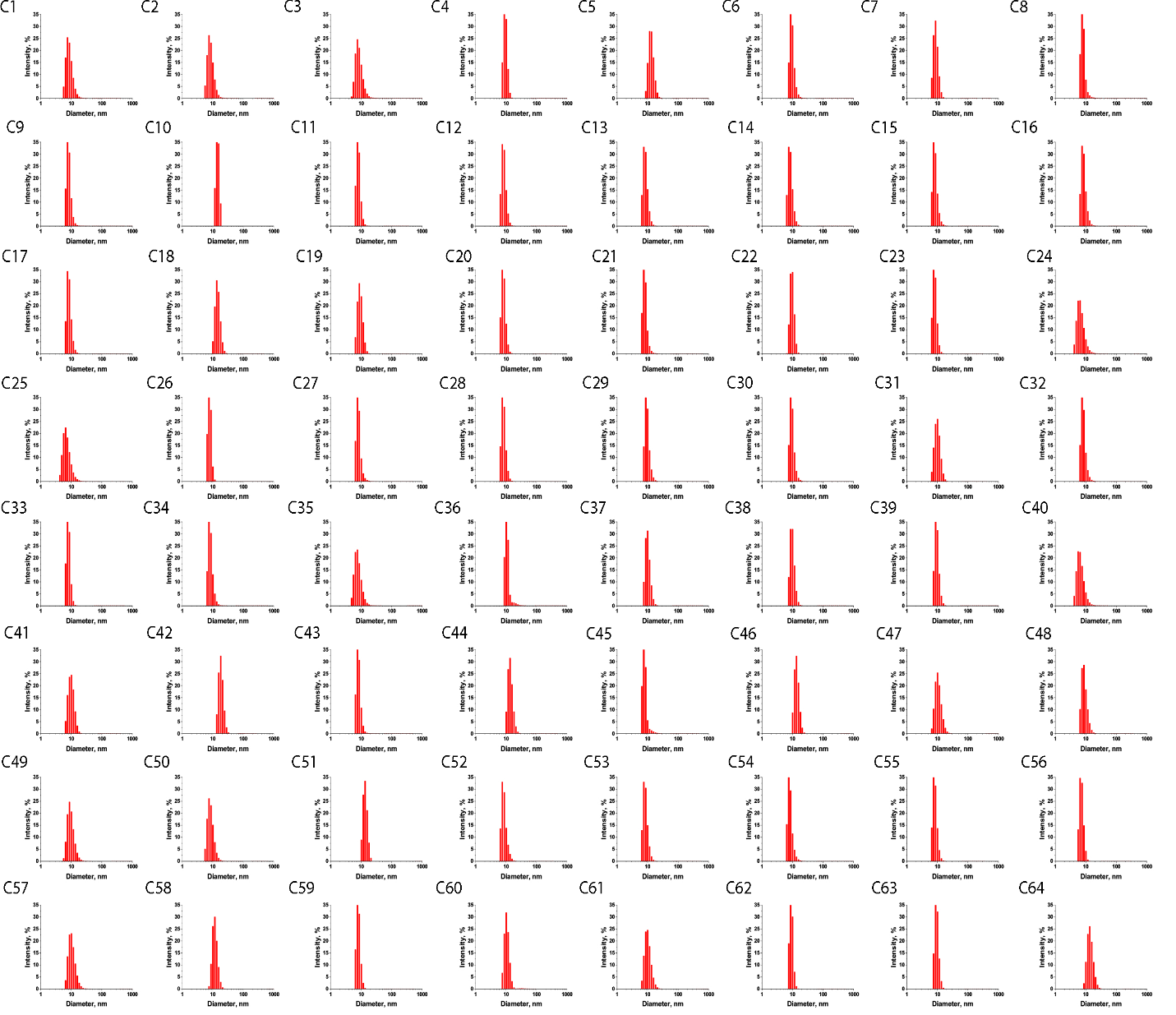


**Supplemental Figure S4.** Representative DLS spectra of nucleic acid nanoparticles (NANPs). DLS analysis was performed on a series of 64 cuboidal (C1–C64) NANPs. Size distributions from the DLS measurements indicate the hydrodynamic diameters of each structure. The polydispersity indices for cuboidal structures were below 0.3, indicating a higher monodisperse population.


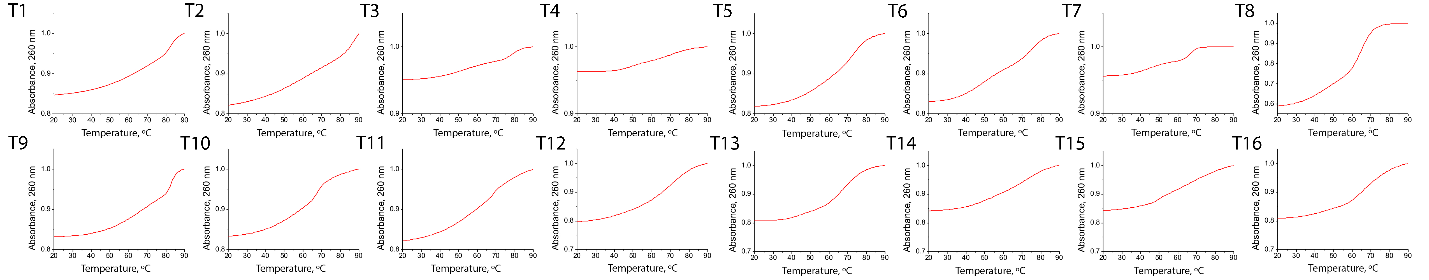


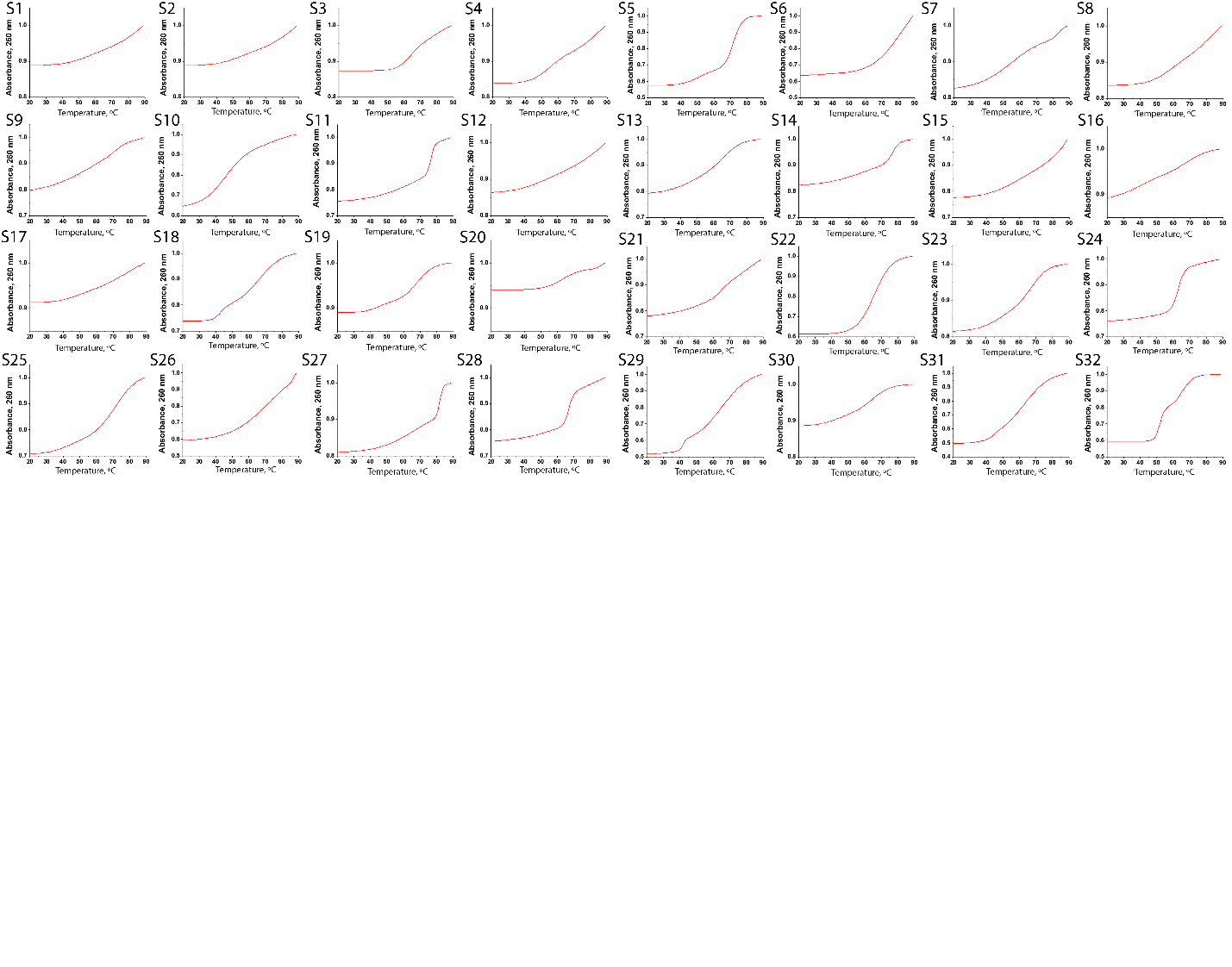


**Supplemental Figure S5.** Representation UV-melting profiles of NANPs. Thermal melts were collected on a series of assembled polygonal structures, including a set of 16 triangular (T1–T16) and 32 square (S1–S32) NANPs. Absorbance measurements were recorded from 20°C to 90°C at 1°C per minute intervals to monitor melting transitions for each structure. All polygonal structures display a characteristic sigmoidal curve reflecting differences in thermal stability among the geometries.

**
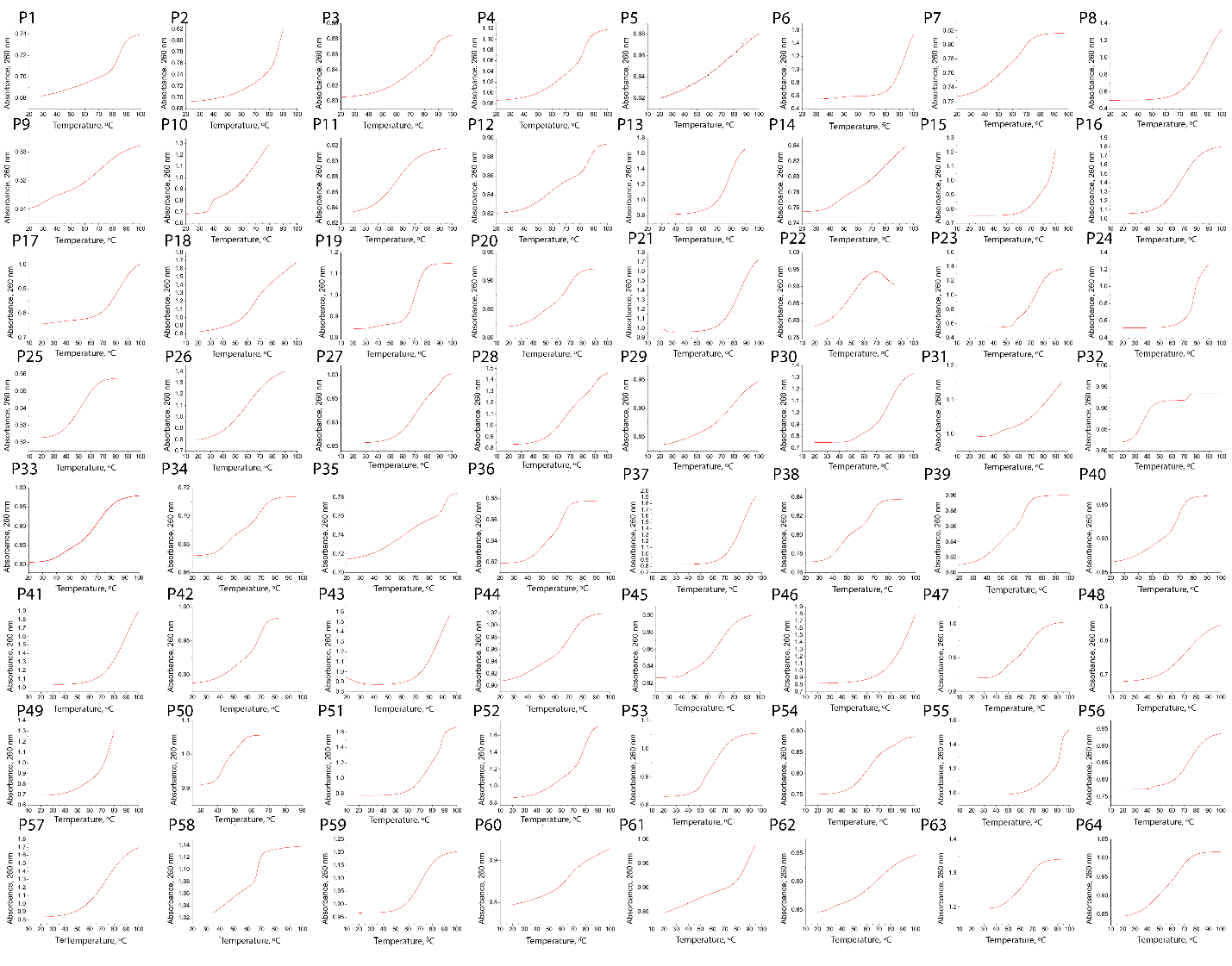
**

**Supplemental Figure S6.** Representation UV-melting profiles of NANPs. Thermal melts were collected on a series of 64 assembled pentagonal (P1–P64) NANPs. Absorbance measurements were recorded from 20°C to 90°C at 1°C per minute intervals to monitor melting transitions for each structure. All polygonal structures display a characteristic sigmoidal curve reflecting differences in thermal stability among the geometries.


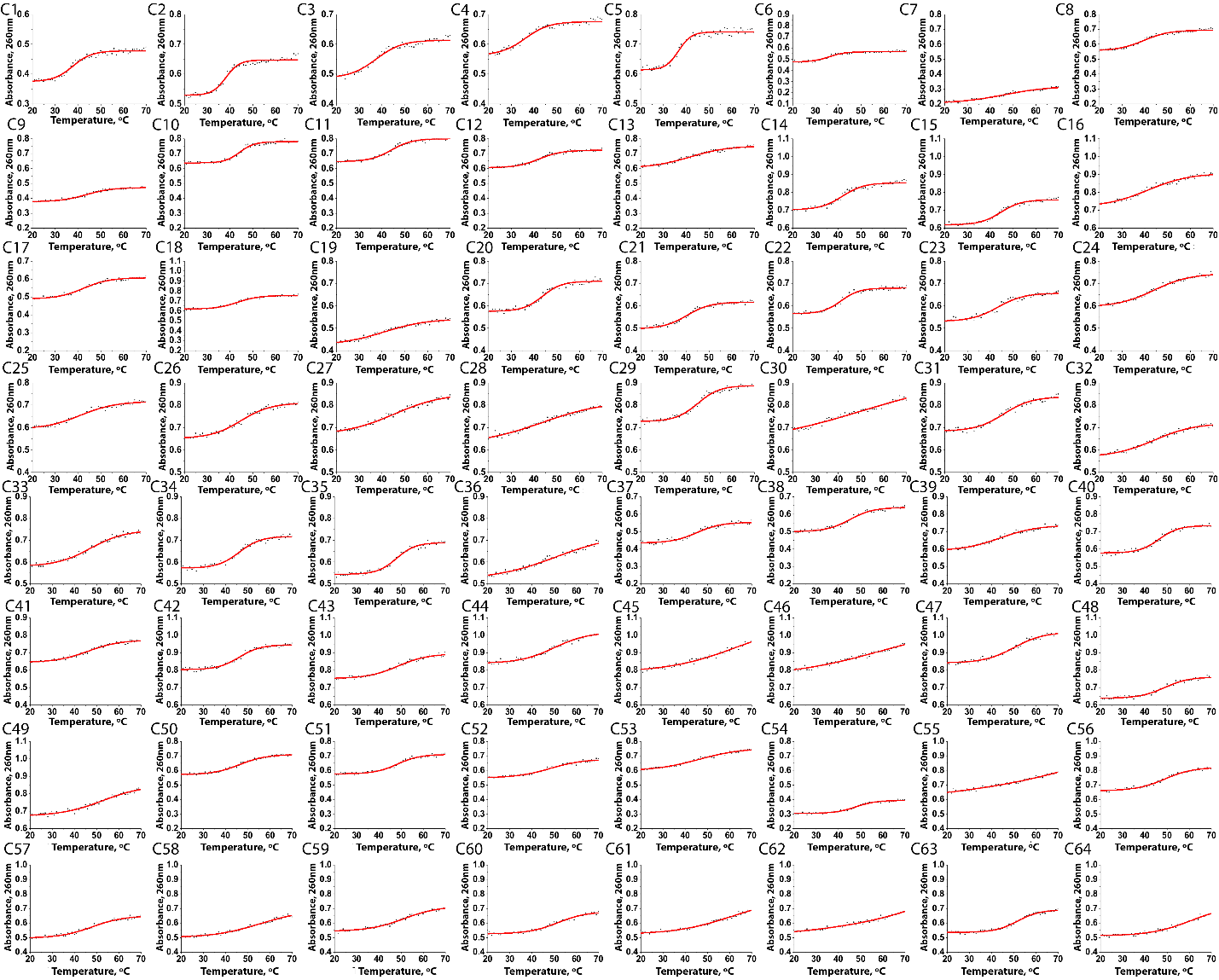


**Supplemental Figure S7.** Representation UV-melting profiles of NANPs. Thermal melts were collected on a series of 64 assembled cuboidal (C1–C64) NANPs. Absorbance measurements were recorded from 20°C to 90°C at 1°C per minute intervals to monitor melting transitions for each structure. All cuboidal structures display a characteristic sigmoidal curve reflecting differences in thermal stability among the geometries.


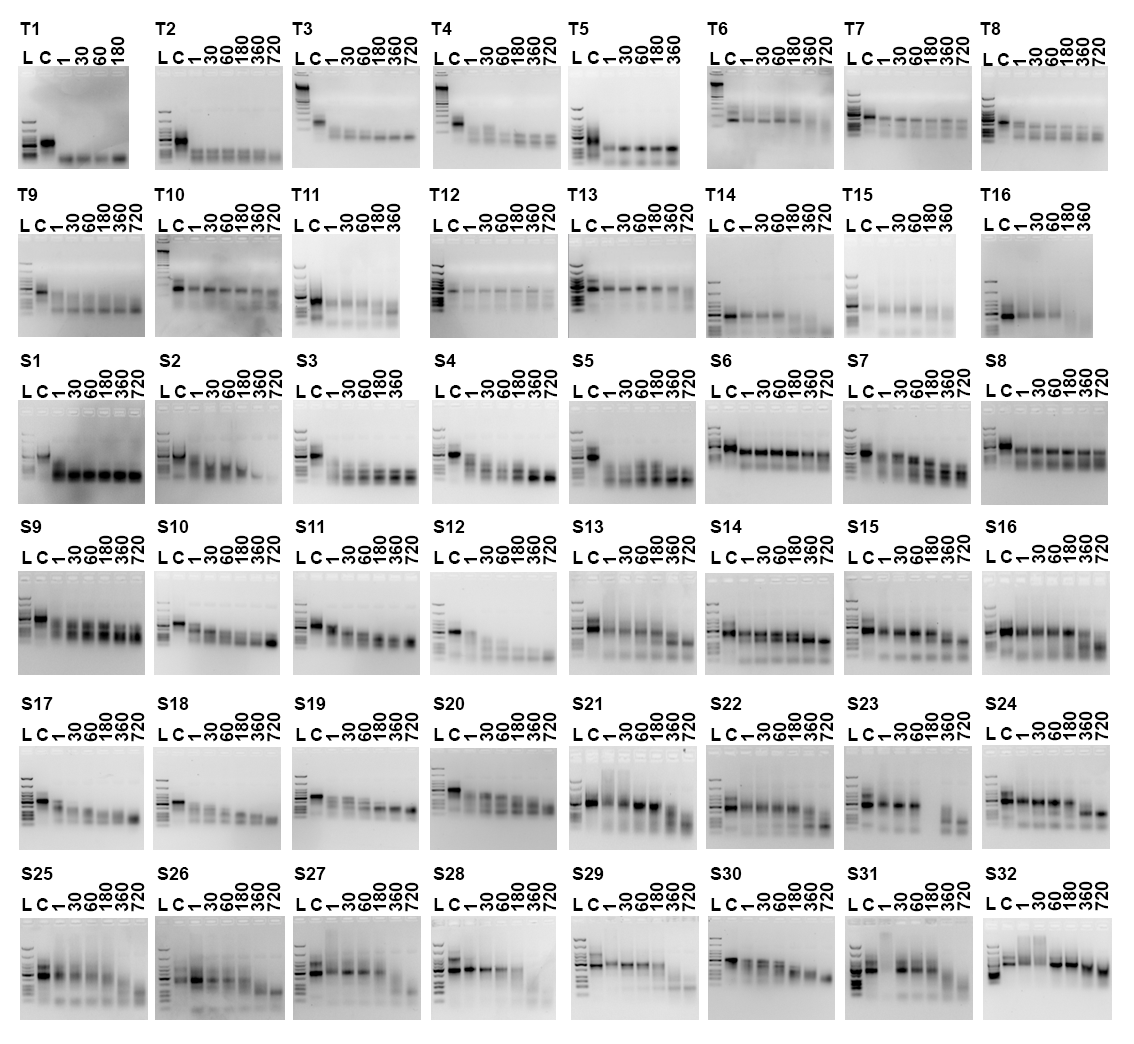


**Supplemental Figure S8.** Stability profiles of nucleic acid nanoparticles (NANPs) in 20 % FBS evaluated by 0.8% agarose gel electrophoresis. Panels show stability profiles for triangular (T1–T16) and square (S1–S32). Lane L represents the Low Range Molecular Weight DNA ladder (NEB), and Lane C is the control NANP sample, untreated with FBS. All other lanes contain sample fractions collected at specific time points, indicated in minutes.


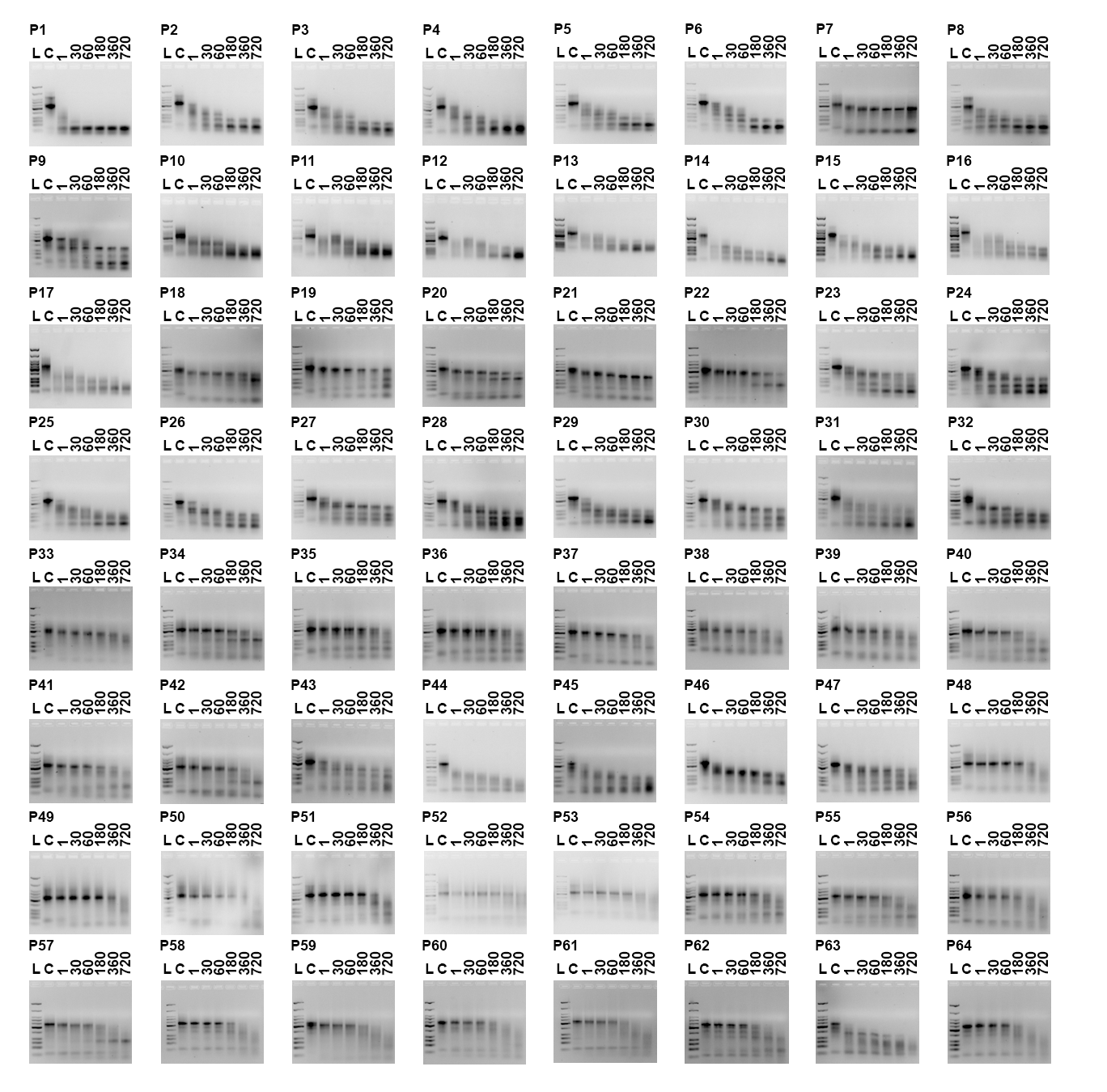


**Supplemental Figure S9.** Stability profiles of nucleic acid nanoparticles (NANPs) in 20 % FBS evaluated by 0.8% agarose gel electrophoresis. Panels show stability profiles for pentagon (P1–P64) NANPs. Lane L represents the Low Range Molecular Weight DNA ladder (NEB), and Lane C is the control NANP sample, untreated with FBS. All other lanes contain sample fractions collected at specific time points, indicated in minutes.

**
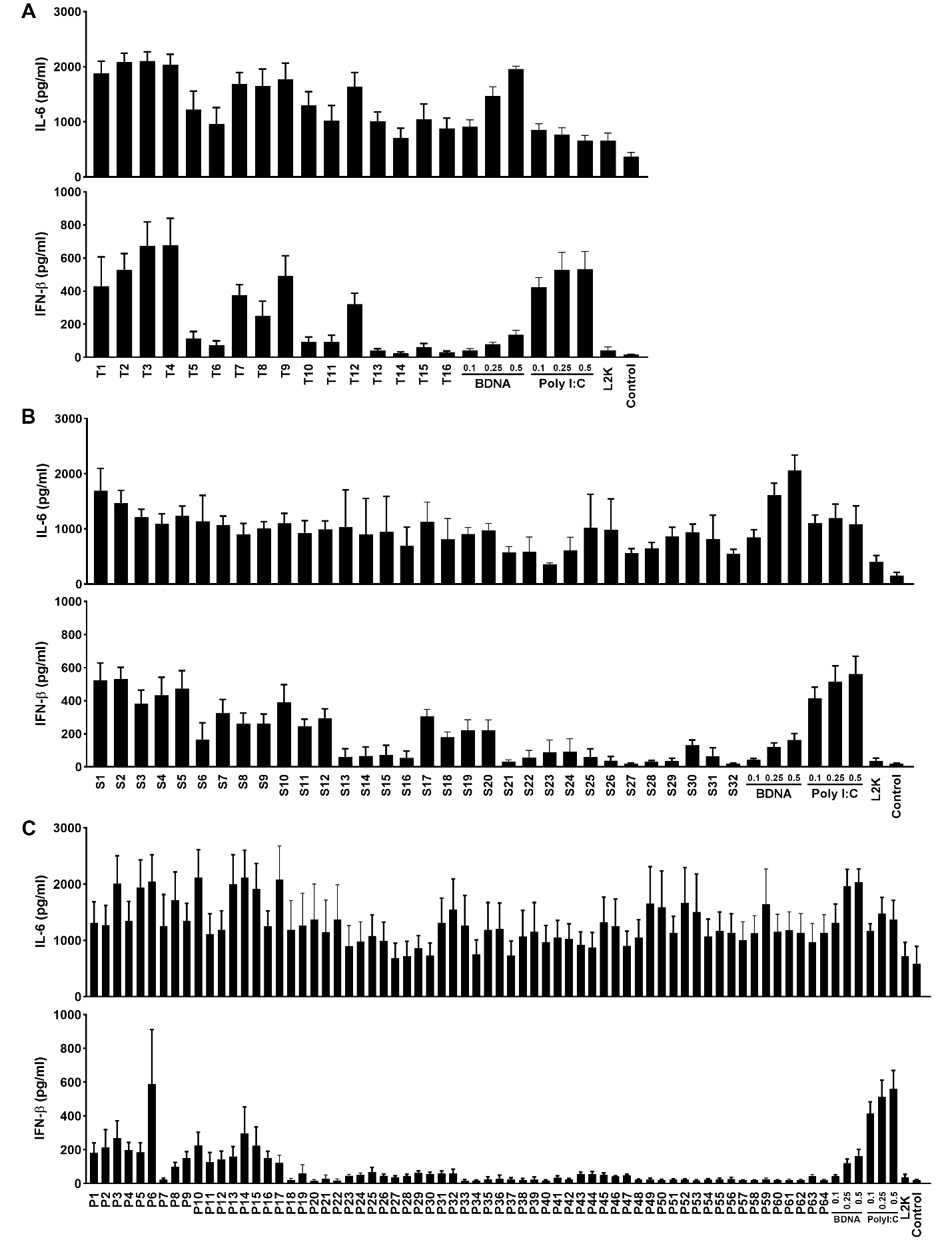
Supplemental Figure S10**. Human microglia were transfected with carrier alone (lipofectamine 2000, L2K), 0.1- 0.5 ug/mL BDNA, 0.1- 0.5 ug/mL Poly I:C, or 5nM triangle (A), square (B), or pentagon (C) NANPs. Cell supernatants collected 24 h post transfection. IL-6 and IFN-*β* production was quantified using specific capture ELISAs. Data are expressed as the mean ± SEM for a minimum of three independent replicates

**
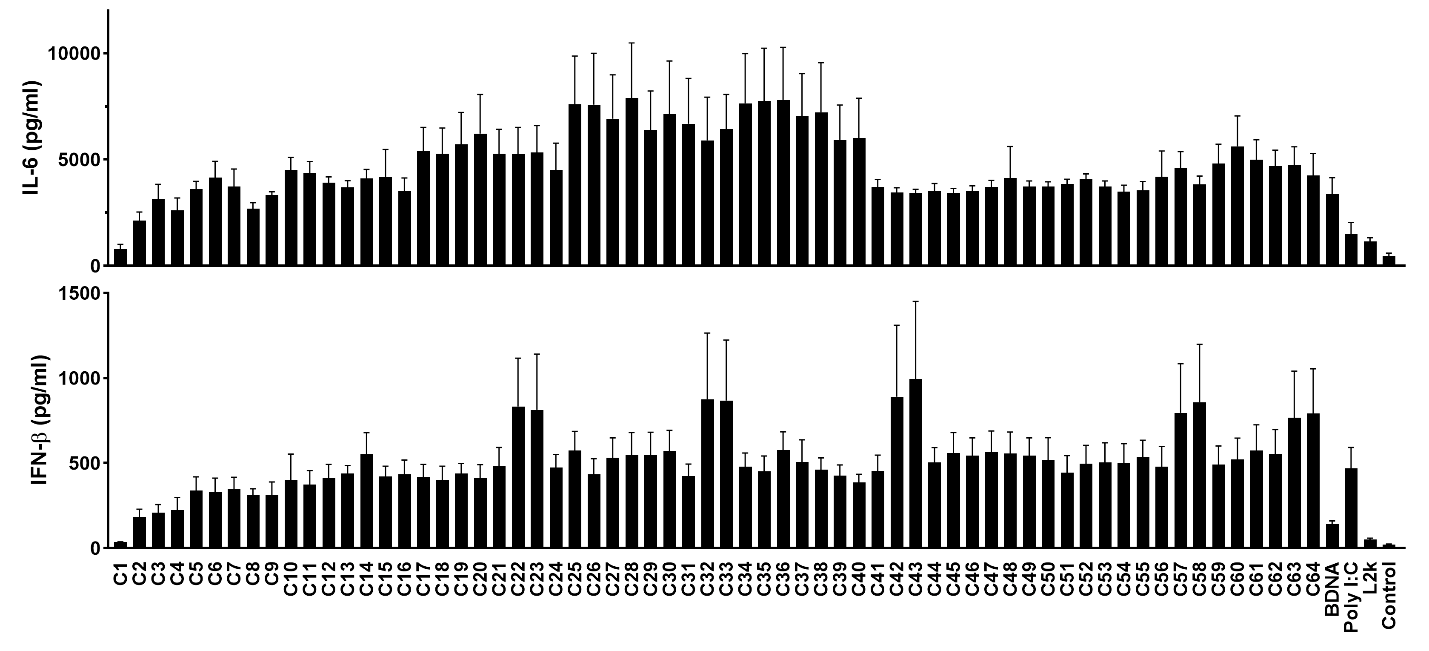
Supplemental Figure S11**. Human microglia were transfected with carrier alone (lipofectamine 2000, L2K), 0.1- 0.5 ug/mL BDNA, 0.1- 0.5 ug/mL Poly I:C, or 5nM cube NANPs. Cell supernatants collected 24 h post transfection. IL-6 and IFN-*β* production was quantified using specific capture ELISAs. Data are expressed as the mean ± SEM for a minimum of three independent replicates.

**
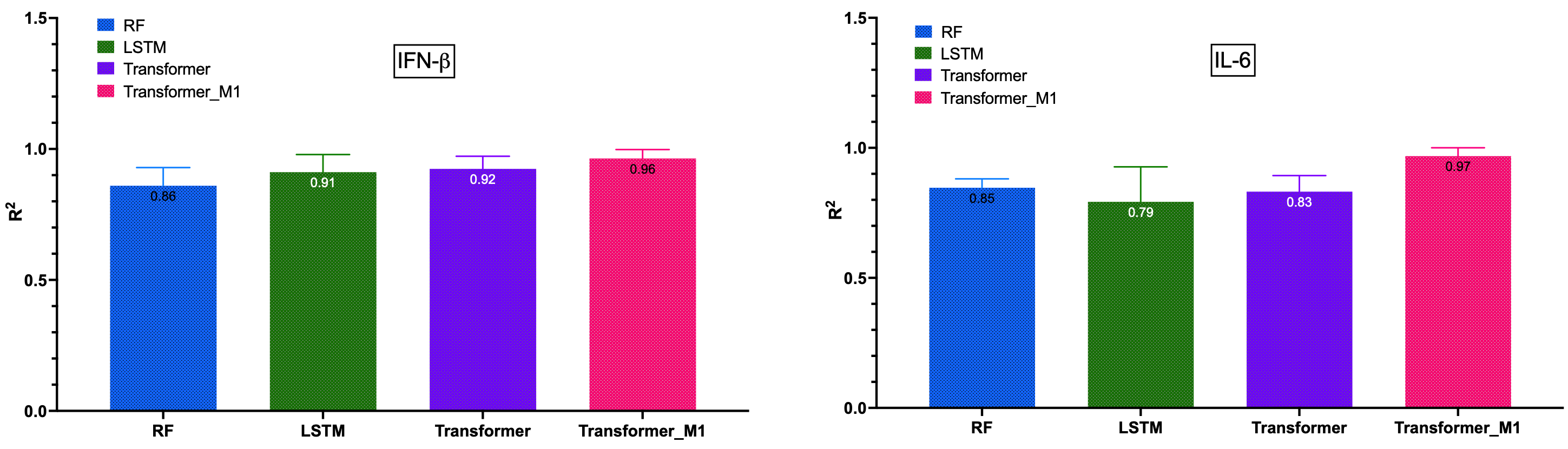
**

**Supplemental Figure S12**. Average predictive performance (R²). Bars show mean R² across five-fold cross-validation repeated ten times (n = 50); error bars indicate the standard deviation across the 50 evaluations. Legend: Random Forest (blue), LSTM (green), Transformer (purple), Transformer_M1 (magenta). Detailed statistics and pairwise comparisons are reported in SI Table S5A-B.


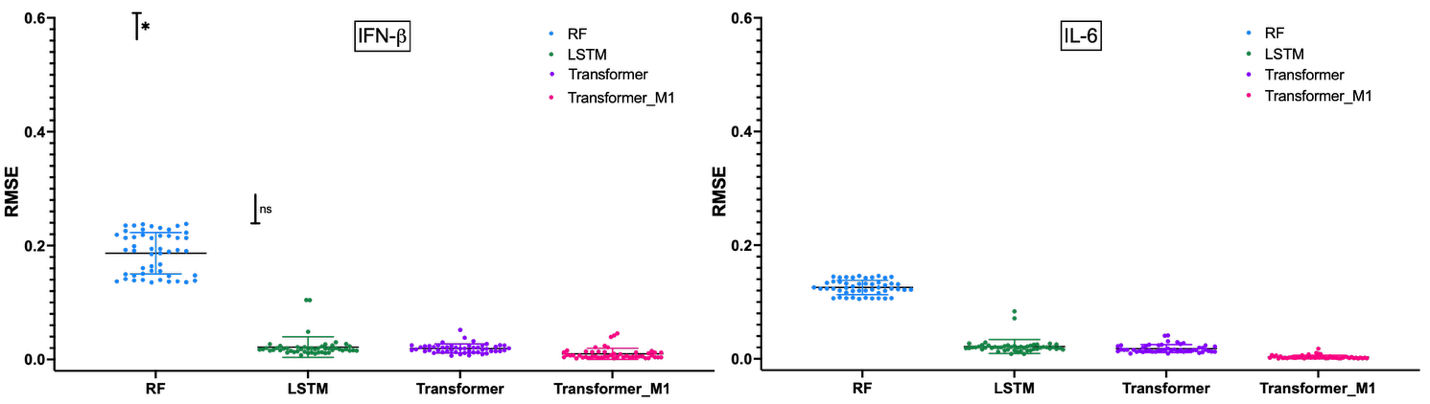


**Supplemental Figure S13**. Average predictive error (RMSE). Bars show mean RMSE across five-fold cross-validation repeated ten times (n = 50); error bars indicate the standard deviation across the 50 evaluations. Legend: Random Forest (blue), LSTM (green), Transformer (purple), Transformer_M1 (magenta). Detailed statistics and pairwise comparisons are reported in SI Table S5A-B.


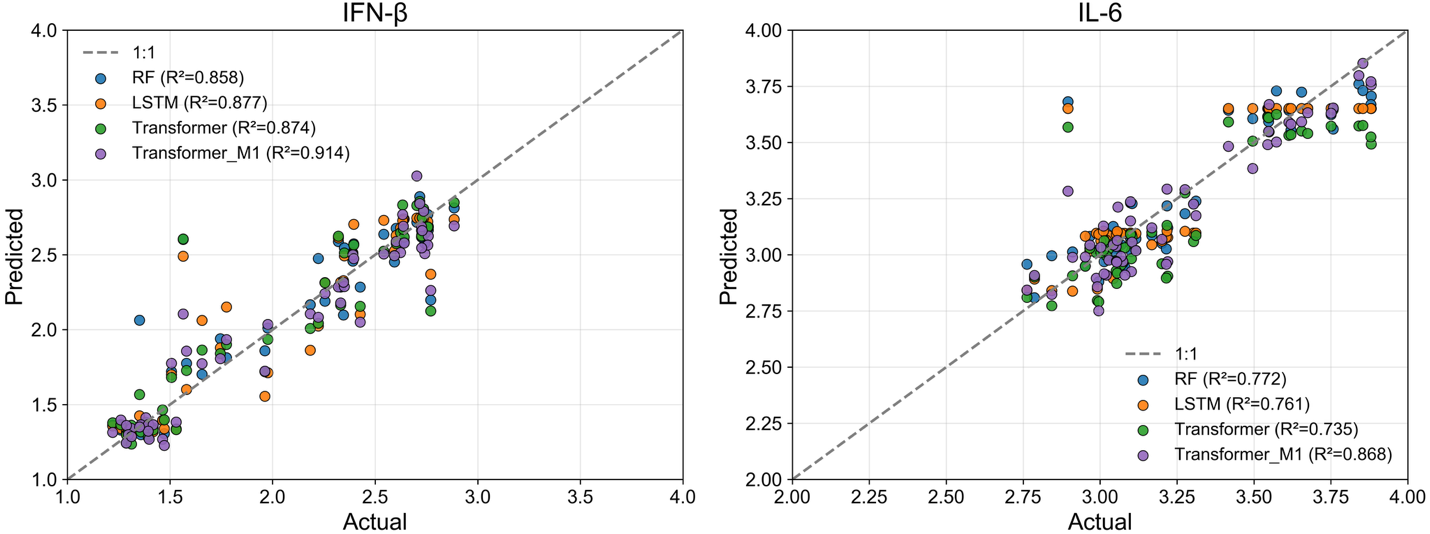


**Supplementary Figure S14.** Actual vs predicted cytokine responses on the external test set (two panels).

Scatter plots show observed (x-axis) versus model-predicted (y-axis) values for IFN-β (left) and IL-6 (right). Points are individual test-set constructs (n = 53) Colors denote models: Random Forest, LSTM, Transformer (no augmentation), and transformer_M1. The dashed line is the 1:1 reference. Targets were log10-transformed for modeling; axes reflect the log scale. Detailed statistics and pairwise comparisons are reported in SI Table S7A-B.

Performance summary (external test, n = 53). Among the four approaches, transformer_M1 performed best on both endpoints—IFN-β R² = 0.914 and IL-6 R² = 0.868—followed by the transformer without augmentation (IFN-β R² = 0.874; IL-6 R² = 0.735) and the LSTM (IFN-β R² = 0.877; IL-6 R² = 0.761). The RF baseline trailed (IFN-β R² = 0.858; IL-6 R² = 0.772). Trend-line fits (predicted vs actual) show transformer_M1 closest to the ideal (slope ≈ 1, intercept ≈ 0), indicating the lowest bias among the compared models.
